# Proteogenomic characterization identifies clinical subgroups in *EGFR* and *ALK* wild-type never-smoker lung adenocarcinoma

**DOI:** 10.1038/s12276-024-01320-0

**Published:** 2024-09-19

**Authors:** Hyondeog Kim, Wonyeop Lee, Youngwook Kim, Sang-Jin Lee, Wonyoung Choi, Geon Kook Lee, Seung-Jin Park, Shinyeong Ju, Seon-Young Kim, Cheolju Lee, Ji-Youn Han

**Affiliations:** 1 Graduate School of Cancer Science and Policy, National Cancer Center, Goyang, Gyeonggi-do Republic of Korea; 2 Anticancer Resistance Branch, Research Institute of National Cancer Center, Goyang, Gyeonggi-do Republic of Korea; 3 Division of Cancer Data Science, Research Institute of National Cancer Center, Goyang, Gyeonggi-do Republic of Korea; 4 Immuno-oncology Branch, Research Institute of National Cancer Center, Goyang, Gyeonggi-do Republic of Korea; 5 Cancer Molecular Biology Branch, Research Institute of National Cancer Center, Goyang, Gyeonggi-do Republic of Korea; 6 Cancer Diagnostics Branch, Research Institute of National Cancer Center, Goyang, Gyeonggi-do Republic of Korea; 7 Korea Research Institute of Bioscience and Biotechnology, Daejeon, Republic of Korea; 8 Department of Bioscience, University of Science and Technology, Daejeon, Republic of Korea; 9 Chemical & Biological Integrative Research Center, Korea Institute of Science and Technology, Seoul, Republic of Korea; 10 Korea Bioinformation Center, Korea Research Institute of Bioscience and Biotechnology, Daejeon, Republic of Korea; 11 Division of Bio-Medical Science & Technology, KIST School, Korea University of Science and Technology, Seoul, Republic of Korea

**Keywords:** Functional clustering, Cancer genomics, Transcriptomics, Proteome informatics

## Abstract

Patients with lung adenocarcinoma who have never smoked (NSLA) and lack key driver mutations, such as those in the *EGFR* and *ALK* genes, face limited options for targeted therapies. They also tend to have poorer outcomes with immune checkpoint inhibitors than lung cancer patients who have a history of smoking. The proteogenomic profile of nonsmoking lung adenocarcinoma patients without these oncogenic driver mutations is poorly understood, which complicates the precise molecular classification of these cancers and highlights a significant area of unmet clinical need. This study analyzed the genome, transcriptome, and LC‒MS/MS-TMT-driven proteome data of tumors obtained from 99 Korean never-smoker lung adenocarcinoma patients. NSLA tumors without *EGFR* or *ALK* driver oncogenes were classified into four proteogenomic subgroups: proliferation, angiogenesis, immune, and metabolism subgroups. These 4 molecular subgroups were strongly associated with distinct clinical outcomes. The proliferation and angiogenesis subtypes were associated with a poorer prognosis, while the immune subtype was associated with the most favorable outcome, which was validated in an external lung cancer dataset. Genomic-wide impacts were analyzed, and significant correlations were found between copy number alterations and both the transcriptome and proteome for several genes, with enrichment in the ERBB, neurotrophin, insulin, and MAPK signaling pathways. Proteogenomic analyses suggested several targetable genes and proteins, including *CDK*s and *ATR*, as potential therapeutic targets in the proliferation subgroup. Upregulated cytokines, such as *CCL5* and *CXCL13*, in the immune subgroup may serve as potential targets for combination immunotherapy. Our comprehensive proteogenomic analysis revealed the molecular subtypes of *EGFR-* and *ALK*-wild-type NSLA with significant unmet clinical needs.

## Molecular subtypes in non-smoker lung cancer: proteogenomic insights

Lung cancer is the leading cause of cancer deaths worldwide, with increasing cases in non-smokers, particularly Asian women. This research investigates lung adenocarcinoma in non-smokers who don’t have common genetic changes, using a multi-omics approach. The study involved 99 patients, specifically those without typical EGFR or ALK mutations, to better understand the disease at a molecular level and find new treatments. The study shows the variety within non-smoker lung cancers and suggests that different groups may need specific treatments. Understanding the molecular types of lung adenocarcinoma in non-smokers can lead to better, personalized treatments and improved health outcomes. This research could lead to more effective treatments for non-smoker lung cancer, potentially improving survival and quality of life for this growing patient group. This summary was initially drafted using artificial intelligence, then revised and fact-checked by the author.

## Introduction

Lung cancer is the leading cause of cancer death, with an annual incidence of approximately 2 million cases worldwide^1^. While smoking remains the primary cause of lung cancer, never-smoker lung cancer is an increasingly concerning public health issue, particularly in Asian female populations, with its incidence steadily rising worldwide over the last several decades^2^. Most of these cancers exhibit a biologically simple cancer network, often characterized by a single predominant oncogenic driver, such as an *EGFR* or *ALK* mutation. Blockade of these mutations by tyrosine kinase inhibitors (TKIs) typically results in an initial clinical response lasting several months^2–4^. However, a considerable proportion of never-smokers with lung cancer lack these identifiable major driver mutations, which poses a challenge for the application of targeted therapies.

Recent clinical trials have reported markedly improved survival in lung cancer patients receiving immune checkpoint inhibitors (ICIs) either as monotherapy or in combination with cytotoxic chemotherapy^5–7^. While ICIs generally improve clinical outcomes in current- and former-smokers with lung cancer, never-smokers typically exhibit a lower tumor mutation burden (TMB) and a reduced presence of immune components, leading to limited clinical benefit from ICIs^8^. Never-smoker non-small cell lung cancer (NSCLC) patients often exhibit resistance to ICIs, even those with high PD-L1 expression levels. Despite the use of the TMB as an ICI efficacy biomarker, its clinical implementation faces challenges such as the need to acquire adequate tissue for sequencing, lengthy turnaround times, and high costs^8^. Moreover, an optimal cutoff value for the TMB as a predictive biomarker has yet to be determined^9^.

In patients with never-smoker lung adenocarcinoma (NSLA), targetable driver oncogenes are enriched, and these patients are treated with selective TKIs rather than ICIs^3,9,10^. Given the inferior efficacy of ICIs in treating NSLA, therapeutic options are limited for this population in the absence of identifiable targetable driver oncogenic mutations.

The uncertain applicability of ICIs and limited options for targeted therapeutics prompted us to examine the fundamental molecular characteristics of NSLA patients lacking major driver mutations. The strong predictive association of driver mutations with clinical responses in patients with driver-positive NSLA suggests that a holistic understanding of NSLA without major driver mutations can be achieved through integrated multiomics studies. Previous research has extensively described the molecular landscape of never-smoker lung cancer^11–17^. These studies identified frequent alterations in *EGFR* and *TP53* and classified the samples based on their molecular characteristics^11–14,16,17^. However, there has been a scarcity of proteogenomic research addressing never-smoker lung cancer patients who lack *EGFR* and *ALK* oncogenic mutations. The favorable clinical responses to targeted therapeutics observed in lung cancers with driver mutations underscore the need for a deeper understanding of NSLAs without these mutations through a comprehensive multiomics approach. In our study, we selected patients who were diagnosed with NSLA and had wild-type *EGFR* and *ALK* and subjected them to extensive genomic, transcriptomic, and proteomic analyses. Our integrated multiomics analysis revealed distinct molecular subgroups associated with divergent clinical outcomes, providing critical insights for the refined stratification of these patients and the development of personalized treatment strategies for this particular lung cancer population.

## Materials and methods

### Data collection

RNA sequencing and proteome data were generated from lifelong never-smokers who were diagnosed with lung adenocarcinoma and surgically treated at the National Cancer Center (Goyang, Republic of Korea). Tumors and normal tissues adjacent to the tumors (NAT) were obtained from surgically resected samples. The tissues were cut into cubes, stored in cryomolds, covered with cryogel in optimal cutting temperature compound (OCT, Histolab Products AB, Göteborg, Sweden), frozen in isopentane/dry ice (−120 °C), and transferred to a low-temperature freezer (−80 °C) for long-term storage. The samples were used in accordance with the ethical rules of the National Cancer Center and the Korean Biobank.

### Screening of known drivers from formalin-fixed paraffin-embedded (FFPE) tissues

Genomic DNA was purified using the QIAamp^®^ DNA FFPE Tissue Kit (Qiagen, Hilden, Germany) and quantified using a Nanodrop^™^ 2000 (Thermo Fischer Scientific, Inc., Waltham, MA, USA). The DNA samples that passed quality control were further analyzed by targeted sequencing of known driver genes using a HiSeq 2500 system (Illumina, Inc., San Diego, CA, USA). A list of the genes is provided in Supplementary Table 4.

### DNA and RNA preparation, library construction, and sequencing

Genomic DNA and total RNA were extracted using the AllPrep DNA/RNA Mini Kit (Qiagen) and assessed with an ND-1000 spectrophotometer (Thermo Fisher Scientific, Inc.). RNA integrity was evaluated using a 2100 Bioanalyzer (Agilent Technologies, Inc., Germany). For whole-genome sequencing (WGS) and RNA sequencing library preparation, the TruSeq DNA Library Prep Kit and TruSeq Stranded Total RNA Sample Preparation Kit (Illumina, Inc.) were utilized. Whole-genome sequencing was performed with average depths of 48x for tumor samples and 44x for normal samples for WGS. The prepared libraries were subjected to paired-end sequencing with NovaSeq 6000 and HiSeq 4000 systems (Illumina, Inc.). Sequencing read quality was assessed with FastQC (v0.11.7) for primer/adapter sequence contamination. DNA and RNA FASTQ sequences were trimmed using TrimGalore, with a threshold of average sequence quality >30 according to previous proteogenomic research^12^. Trimmed reads were aligned to the human reference genome GRCh38 using BWA-MEM v0.7.17 for DNA and STAR (STAR-2.7.0)^18^ for RNA. SAM files were converted to BAM files with SAMtools (v1.3.1), followed by duplicate read removal using Picard MarkDuplicates. GATK (v3.7.0.0) tools, including RealignerTargetCreator and IndelRealigner, were used for misalignment correction. Base quality scores were adjusted with BaseRecalibrator, and the final analysis was performed with PrintReads, resulting in recalibrated merged BAM files sorted in coordinate order. Variant calling was performed using MuTect2 with cosmic coding mutants, cosmic noncoding mutants, and dbsnp v146 and annotated with ANNOVAR (2018-04-16 version). Redundant genes were combined to calculate the average RPKM, and RNA expression data were normalized as previously described^12^.

### Peptide fractionation, TMT-10 labeling, and LC‒MS/MS-based proteomics analysis methods

Protein extraction, TMT-10 peptide labeling, and peptide fractionation via basic pH reverse-phase liquid chromatography (bRPLC) were conducted using an Agilent 1290 Infinity LC System (Agilent Technologies). Chromatography was performed with an XBridge BEH130C18 column (Waters Corporation) with dimensions of 4.6 μm inner diameter × 250 mm length, a pore size of 130 Å, and a particle size of 3.5 μm at a flow rate of 0.5 mL/min. The mobile phases consisted of 10 mM NH4HCO2 (pH 10) for phase A and 10 mM NH4HCO2 (pH 10) in 90% CAN for phase B. Peptides dissolved in 110 μL of phase A were injected into a 100 μL sample loop. The gradient ranged from 2 to 75% B over 80 min.

Fractionation involved collecting 84 tubes (each 0.8 min) pooled into twelve concatenated fractions based on arithmetic sequences; 5% of each fraction was used for global proteome analysis and dried, while the remaining 95% was combined into 12 fractions. The flow-through fractions from bRPLC were merged into one fraction for phosphopeptide enrichment and were subsequently dried.

The phosphopeptides were subjected to metal oxide affinity chromatography using titanium dioxide beads (10 μm) (Titansphere Phos-TiO Bulk). Peptides and TiO2 beads were separately preincubated in a solution containing 3.45 M lactic acid (302 mg/mL), 60% ACN, and 0.3% TFA and then combined and incubated. Phosphopeptide-enriched beads were collected by centrifugation, and the unbound supernatants were pooled for double TiO2 enrichment. The beads were washed, loaded onto a C8-plugged tip, eluted with 1.5% NH4OH and 5% pyrrolidine, acidified, desalted using graphite spin columns, dried, resuspended in 0.1% formic acid, and loaded onto a C18 trap column (2 cm × 100 μm) using an Eksigent nanoLC-ultra 1D plus system at 5 μL/min. Subsequently, the peptides were separated on an EASY-Spray column (50 cm × 75 μm) using a gradient of 4–32% acetonitrile with 0.1% formic acid over 150 min, followed by 32–80% acetonitrile over 10 min at a flow rate of 250 nL/min.

HPLC was coupled to a Q Exactive mass spectrometer operating in data-dependent acquisition mode. Full-scan MS spectra (m/z 400–2000) were acquired at 70,000 resolution, followed by tandem MS of the twelve most intense ions with charge states of 2–5. The ionization parameters included a spray voltage of 1.9 kV, a capillary temperature of 275 °C, and an s-lens level of 50.0. For MS/MS acquisition, the following parameters were used: resolution = 35,000, automatic gain control target = 5e^4^, isolation width = 1.2 m/z, maximum injection time = 120 ms, collision energy = 35%, dynamic exclusion = 60 s, and ion selection threshold = 2.5e^4^.

### Database customization and annotation of somatic and germline variants

Somatic and germline variants were annotated based on Hg38 RefSeq, focusing on protein-altering events such as nonsynonymous SNVs, frameshift and nonframeshift indels, stop losses, and novel junctions. Using the R package “customProDB” (v1.28.0)^19^, a customized database for each tumor sample was constructed using germline and somatic variants obtained from corresponding WGS data. Variants from nine samples in a TMT set were combined, and redundancies were merged into a single FASTA entry. Following somatic variant annotation, neoantigen identification was performed using pVAC-Seq^20^. These data were processed through a pipeline in which epitopes were predicted, sequencing-based information was integrated, and neoantigen candidates were filtered.

### Measurement of tumor purity and ploidy

The tumor and normal paired BAM files from WGS data were prepared for analysis using the sequenza-util tool, employing a window size of 50 and the hg38 reference genome assembly. Following preprocessing, the resulting seqz files were subjected to in-depth analysis using the Sequenza algorithm^21^ for measuring tumor purity and ploidy.

### Analysis of the correlation between immune genes and scores

Immune infiltration rates were calculated from immune scores using the ESTIMATE algorithm^22^ to explore the intricate relationships within the TME and to determine how changes in immune scores correlated with variations in the expression of these critical immunomodulatory factors (Supplementary Table 11).

### Analysis of mutation significance and signatures

The MutSigCV algorithm (v.1.3.5)^23^ was used to discern significantly mutated genes in our cohort. The default parameters of three inputs, coverage table file, covariate table file and mutation type dictionary were used to run MutSigCV, which utilizes a statistical framework to evaluate the observed mutation patterns against background mutation rates while considering relevant covariates such as sequence context and replication timing.

The “sigminer (v.2.2.0)^24^” R package was used to determine mutational signatures in each sample, including the age signature (clock-like signatures: signature 1 and 5), microsatellite instability (MSI) signature (signatures 14, 15, and 20) and APOBEC signature (signatures 2 and 13). Full genomic sequences for *Homo sapiens* (UCSC genome hg38) were used as a reference genome for mutational analysis, with default NMF clustering parameters.

### Subgroup classification

Investigation of the tumor microenvironments (TMEs) and characterization of tumor intrinsic properties were performed by analyzing transcriptome and proteome data with signature genes obtained from previous studies^22,25,26^, which comprises 31 gene sets related to the TME and malignant cell properties. The full list of signature genes employed in the clustering analyses is provided in Supplementary Table 5. The enrichment of signature genes was determined via single-sample gene set enrichment analysis (ssGSEA) using the ‘GSVA (v.1.46.0)’ R package. Pathway gene set enrichment results from the transcriptome and proteome data were combined into one matrix, clustered using Euclidean distance calculations and the complete method, and then hierarchically split into four groups. The top quartile of patients (0.75, Q3) with a high proliferation signature was labeled the proliferation-high subgroup, aligning with previous findings^12–17,26^. The remaining patients were further subdivided, first into a T-cell signature-high characteristic group designated the immune-high subgroup. The remaining patients were segregated into two subgroups by a hierarchical clustering method. Initially, each data point was treated as an individual cluster and we calculated the pairwise Euclidean distances between all points. The clusters with the smallest maximum pairwise distance were iteratively merged, updating the distance matrix at each step to reflect the new clusters. This process continued until all points were merged into a single cluster or until the desired number of clusters was achieved. The resulting hierarchy of clusters was visualized using a heatmap with gene set enrichment and annotated based on their characteristics. Pathway clustering was performed using the same algorithm used for the abovementioned clustering. The proportion and distribution of clustering using transcriptome, proteome and integrated data are shown in Supplementary Fig. 1c.

### Survival analysis

The R packages “survival (v.3.5-5)” and “survminer (v.0.4.9)” were used to generate survival probability curves. A multivariate Cox proportional hazard regression model was performed based on sex, age, pathological tumor stage, and subgroup, and their coefficients were calculated.

### Protein correlation network analysis

A total of 2,063 proteins were selected based on variance and correlation score (correlation score > 0.5, Pearson method) and subjected to unsupervised hierarchical clustering using complete linkage (Supplementary Fig. 7). Cluster characterization was conducted through enrichment analysis using hallmark gene sets from the Molecular Signature Database (MSigDB). The protein network layout was created using the ForceAtlas2 algorithm from Gephi, a network visualization platform, balancing linear attraction and repulsion forces through the Barnes–Hut method with a scaling parameter set at 1.0 and a gravity value of 15.0 while preventing overlapping nodes (K-core > 3).

### Identification of the subtype-specific protein‒protein interaction network

Gene selection was conducted using Welch’s *t* test adjusted for multiple comparisons. These genes were utilized as inputs for KEGG human pathway enrichment analysis (Supplementary Tables 9–10, *P* < 0.001) via the R package enrichR (v.3.2) and STRING database (v11.5)^27^ to determine the functional associations within the protein–protein interaction (PPI) network. Reference kinase proteins were selected from a list of 536 human kinases (kinhub.org/kinases.html)^28^, and kinase PPI networks were visualized using an edge score of 0.4.

### Cancer cell vulnerability and drug sensitivity analysis

The CERES scores, which are computational representations of clustered regularly interspaced short palindromic repeats (CRISPR) gene essentiality, were sourced from the Cancer Dependency Map (DepMap), which incorporates CRISPR knockout screens from the Achilles project supplemented with genomic data from the Cancer Cell Line Encyclopedia (CCLE). We selected lung adenocarcinoma cell lines lacking *EGFR* or *ALK* driver mutations and with CERES scores less than −0.5 for vulnerability and dependency. The significance of subtype-specific vulnerabilities was determined using the Wilcoxon rank sum test (*P* < 0.05). For drug sensitivity, we used the PRISM Repurposing dataset, which contains data on 4,688 compounds, 578 cell lines, 23 primary diseases, and 25 lineages and contrasts the log fold change values of compounds against DMSO, which can be accessed through the DepMap portal at https://depmap.org/repurposing^29^.

### Cancer germline antigen (CGA) analysis

A compilation of CGA genes was obtained from a previous study^30^. Among 226 candidate cancer germline genes, those with 100-fold higher expression than the average FPKM value within the cohort were selected. Outliers were further filtered through a threshold of more than 3 standard deviations away from the mean. To facilitate the comparative analysis of the expression of the selected genes, normal tissue TPM data sourced from the Genotype-Tissue Expression (GTEx) project^31^ were utilized and merged with FPKM to TPM converted data from the cohort transcriptomic data.

## Results

### Clinical characteristics

A total of 1597 fresh-frozen tumor samples were collected from patients with NSLA who underwent surgical resection between 2001 and 2018 at the Korean National Cancer Center (KNCC). Among these, 102 samples from tumors without drug-sensitive *EGFR* mutations or *ALK* rearrangements were collected for further analyses (Supplementary Fig. 1a). Genomic data was used for tumor purity estimation, resulting in the exclusion of three samples with low purity (purity < 0.4, Supplementary Fig. 1b). A total of 99 samples were selected as frozen sample sets, and these 99 samples were subsequently used for downstream analyses. The median age of the patients was 63 years (range, 40–85 years), and 87.9% (n = 87) of them were women; 39.4%, 33.3%, and 27.3% of the tumors were stage I, II, and III, respectively.

### Classification of subgroups

**Fig. 1:**
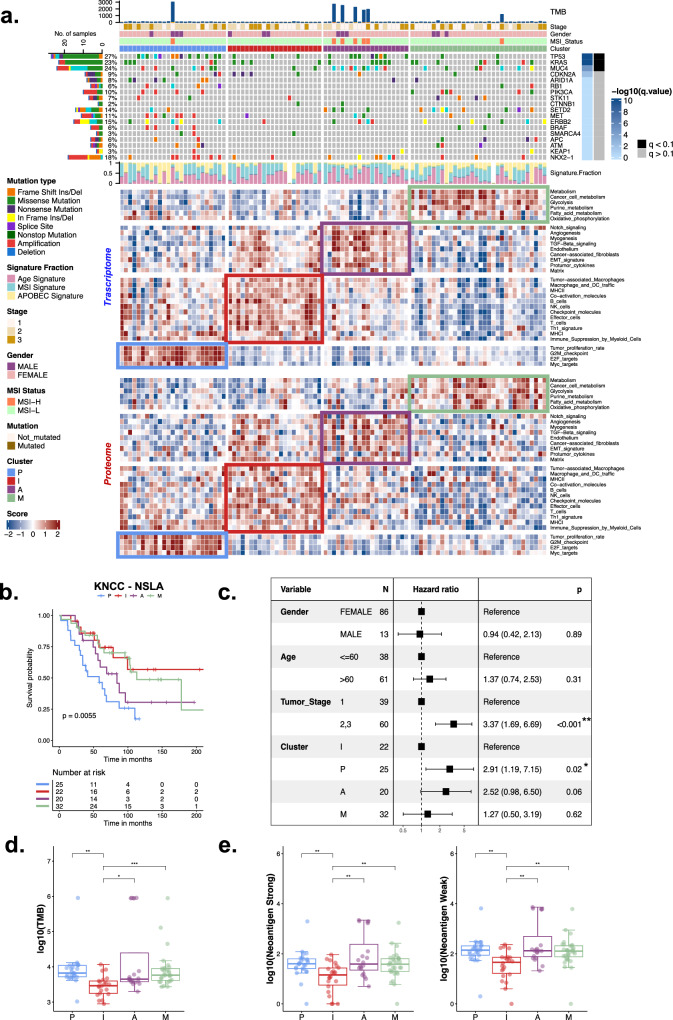
Immune microenvironment and TME proteogenomic profiles of NSLA. **a** Heatmap representation of distinct molecular subgroups in the NSLA cohort. The subgroups are color-coded as follows: proliferation-high (P) subgroup, blue; immune-high (I) subgroup, red; angiogenesis-high (A) subgroup, purple; and metabolism (M) subgroup, green. Mutations are ordered by significance calculated by MutSigCV. Three mutational signatures were found in this cohort: age (pink), MSI (blue) and APOBEC (yellow). **b** Overall survival probability of the molecular subgroups of NSLA (Kaplan–Meier survival analysis with log-rank P value). **c** Cox proportional hazard model multivariate analysis of molecular classification adjusted for clinical factors, including sex, age, and tumor stage and subtype. **d** Boxplot of the TMB and (**e**) amount of weak or strong neoantigen according to molecular subgroup.

The scarcity of proteogenomic research on lung adenocarcinoma in never smokers without *EGFR* or *ALK* mutations has resulted in an oversimplified view of these diseases, treating them collectively as a single, homogeneous, yet undefined entity. To explore the possibility of suitable stratification of molecular subgroups within NSLA without *EGFR* or *ALK* mutations, we employed a set of gene signatures that were shown to represent tumor-intrinsic properties as well as characteristics of the tumor microenvironment from a series of previous pan-cancer studies^25,26^. We employed the gene set signatures, encompassing proliferation and immune enrichment scores (see Supplementary Fig. 1a, “Methods”), to analyze the proteome and RNA data from the present study. These molecular signatures facilitated the stratification of the samples into four subgroups with distinct molecular properties: the proliferation (P), immune (I), angiogenesis (A), and metabolism (M) subgroups (refer to Fig. 1a, “Methods”). The molecular factors employed in the classification demonstrated orthogonal classification at both the transcriptome and proteome levels (Fig. 1a, Supplementary Fig. 2a) and were sufficiently independent of each other to guide the subtyping of NSLA patients with varying degrees of clinical outcomes (Supplementary Fig. 2b, c). Among the subgroups, the P and A subgroups exhibited worse clinical outcomes, while the I subgroup displayed more favorable outcomes (Fig. 1b). Multivariate analysis revealed that the hazard ratio for the P subgroup was 2.91 times greater than that for the I subgroup (*P* < 0.05, Fig. 1c).

We utilized transcriptomic data from The Cancer Genome Atlas (TCGA) for external validation. We first utilized TCGA never-smoker lung adenocarcinomas without *EGFR* or *ALK* driver mutations (n = 46). We observed a general trend toward a poor prognosis in the P and A subgroups and a better prognosis in the I subgroup (*P* = 0.0023, Supplementary Fig. 3a, b). The classification of entire TCGA lung adenocarcinoma data into the four molecular subgroups similarly stratified patients according to prognosis (Supplementary Fig. 3c–e).

### The genomic landscape of the NSLA cohort reveals subtype features

In the present cohort, *TP53* exhibited the highest frequency of genetic alterations, at 26.3%, followed by *KRAS* (20.2%) and *SETD2* (13.1%) (Table 1, Fig. 1a). *EGFR* oncogenic driver mutations and *ALK* rearrangements were not detected in the cohort. *ERBB2* exon 20 insertions were frequently observed within the cohort (6 out of 99, Supplementary Fig. 4a). Although patients with *ERBB2* exon 20 insertions have modest sensitivity to HER2 TKIs, they respond better to combinations of ICIs and HER2 TKIs^32^; thus, the *ERBB2* insertion mutation is considered a viable combinatorial therapeutic target. Mutations in another driver gene, *KRAS*, were detected in 20 samples. *KRAS* G12C is the most common type of *KRAS* mutation in lung cancer and was previously reported to be positively associated with smoking status, while the *KRAS* G12D mutation is more frequently found in nonsmokers^33^. In our cohort, *KRAS* G12V and G12D mutations were the most frequent (10 and 6 out of 99, respectively), and *KRAS* G12C was the least frequent *KRAS* mutation type (3 out of 99), with one case of G13C, corroborating the nonsmoking nature of the cohort. The *KRAS* G12C mutation was distributed with one occurrence per cluster, excluding the I subgroup. In the A subgroup, *KRAS* mutations were detected in six samples, among which five had concurrent *TP53* mutations (*P* = 0.0096). *TP53* gene mutations were most frequent in the P subgroup (48%, *P* = 0.0015) and were almost exclusively found in the P and A subgroups (*P* = 1.8 × 10^−5^). Furthermore, two patients in the P subgroup had mutations in both *TP53* and *RB1* (Fig. 1a), which are associated with worse clinical outcomes^34,35^. Other frequent somatic alterations observed in this subgroup included *ARID1A* mutations (*P* = 0.003). Somatic mutations in *SETD2* were inversely correlated with B-cell activity (Supplementary Fig. 3g, *P* = 0.0006) and did not co-occur with functional oncogenic rearrangements (Fisher’s exact test, *P* = 1). *SETD2* mutations were most frequent in the M and A subgroups (*P* = 0.01, Fig. 1a). *STK11* and *PIK3CA* mutations were almost exclusive to the M subgroup (*P* < 0.05).

**Table 1:** Clinical characteristics of patients with *EGFR* and *ALK* wild-type never-smoker lung adenocarcinoma.

Patient characteristics (N = 99)	N (%)
Gender	
Female	86 (86.9)
Male	13 (13.1)
Age, median (range)	63 (40–85)
Stage	
I	39 (39.4)
II	33 (33.3)
III	27 (27.3)
Genetic alterations	
TP53	26 (26.3)
KRAS	20 (20.2)
G12C	3 (3.0)
G12D	6 (6.1)
G12V	10 (10.1)
G13C	1 (1.0)
SETD2	13 (13.1)
ERBB2	12 (12.1)
Exon 20 insertion	7 (7.1)
ROS1^a^	10 (10.1)
ARID1A	8 (8.1)
CDKN2A	8 (8.1)
MET	5 (5.1)
Exon 14 skipping mutation	3 (3.0)
SRK11	5 (5.1)
RET^b^	5 (5.1)
APC	4 (4.0)
SMARCA4	4 (4.0)
PIK3CA	3 (3.0)
BRAF	2 (2.0)
KEAP1	2 (2.0)
RB1	2 (2.0)

^a^ROS1.

^b^RET fusion rearrangements are drug-sensitive alterations.

We assessed mutation signatures in NSLA samples and found that the 3 signatures encompassing the age-related, microsatellite instability (MSI) and APOBEC mutational signatures (Fig. 1a, Supplementary Table 8) were highly related. As expected, there was no smoking-related mutational signature found in the cohort. The proportion of the APOBEC mutational signature was notably greater in the P and M subgroups than in the I and A subgroups (*P* < 0.00022). Interestingly, despite lacking *TP53* mutations, the tumor mutational burden of the M subgroup was comparable to that of the P and A subgroups, while it was significantly lower in the I subgroup (Fig. 1d). Both strong and weak neoantigen loads were also lower in subgroup I (Fig. 1e). Combining a high immune microenvironment and decreased neoantigen loads may produce an immunologically neutral milieu, suggesting another molecular feature contributing to the limited efficacy of ICI monotherapy in patients with NSLA. Additionally, the I subgroup demonstrated the lowest occurrence of the APOBEC mutational signature (*P* < 0.05). A minority of patients with MSI features were mostly segregated within the A subgroup (*P* = 0.009, Fig. 1a), consistent with previous work highlighting the increased expression of vascular endothelial growth factor (*VEGF*) in MSI-high tumors^36^. MSI-high status was also significantly correlated with a lower percentage of patients with a high tumor copy number alteration (CNA) burden (Supplementary Table 6, *P* < *0.05*).

Our study examined the influence of somatic driver mutations on both the proteome and CNA (Supplementary Fig. 4b–d). Among the frequently mutated genes depicted in Fig. 1a, the presence of *STK11* mutation was associated with the most significant upregulation of cancer development regulatory genes. These genes included *CLUH*, components of the oligomeric Golgi family (COG) 2, 4, and 7, as well as *COPZ1*, *IRS2*, *KRAS*, *MVD*, *NEDD4L*, *PGAM5*, *PNPT1*, *RBM28*, *SEC16A*, *SLC12A7*, *SNX27*, *TFCP2*, *TRAF2*, and *TXNRD2* (Supplementary Fig. 4b). Additionally, the protein abundance of *ERBB2* exhibited a strong positive correlation with the CNA of genes located on chromosome 17 (Supplementary Fig. 4c, d).

### Integrative analysis of copy number alterations in NSLA

We conducted an integrative analysis to explore the genome-wide impact of both cis- and trans-acting copy number alterations (CNAs) on the transcriptome and proteome of the NSLA cohort (Supplementary Fig. 5a, *P* < 0.05). Initially, our analysis focused on the cis-acting CNAs of 7364 genes (Supplementary Fig. 5b). Among these genes, we observed a significant correlation between CNA and the transcriptome for 2974 genes and between CNA and the proteome for 431 genes (*P* < 0.05). Gene set enrichment analysis of these 431 genes revealed several oncogenic signaling pathways, including the ERBB, neurotrophin, insulin, and MAPK pathways. Notably, among these genes, 208 showed a positive correlation between CNA and both the transcriptome and proteome (*P* < 0.01, Supplementary Fig. 5b).

In the analysis of trans-acting CNAs, we identified several broadly affecting CNAs in genes that are potentially relevant to lung cancer, including *PTK7*, *METTL1*, *MSTO1*, *PIGU*, *ITGA6*, and *LPCAT4*. Following the exclusion of RNA and unannotated genes, we focused on investigating the effects of trans-acting *ITGA6*. We identified 262 upregulated and 142 downregulated genes based on their proteomic associations. Among these genes, 135 were found to be highly expressed in tumor samples, and 53 were highly expressed in NAT samples (Supplementary Fig. 5c). Furthermore, among the *ITGA6* trans-affected proteomes, 98 genes, including *ACTN1*, *TMOD2*, *MAP2K1*, *BRAT1*, and *SMAD3*, were associated with a worse prognosis when upregulated.

### Proteogenomic characteristics of the proliferation-high (P) subgroup

Overall, the four molecular subgroups of NSLA without driver mutations are characteristic of these clinico-molecular subgroups, and we further investigated the proteogenomic details of each subgroup. The P subgroup was characterized by elevated cellular division rates and had the poorest prognosis. Tumors in this subgroup contained intermixed immune components, generally decreased immune activity, and exhibited significantly depleted angiogenic activity (Fig. 1a, Supplementary Fig. 2a). Considering the contrasting clinical outcomes exhibited by the immune and proliferation groups (Fig. 1b, c), we sought to identify regulatory factors capable of blocking the inherent proliferation potential while enhancing the surrounding immune activity. An algorithm^37^ was used to search for regulatory factors that explain the differentially expressed genes (DEGs) specific to the subgroup of identified transcription factors, including *E2F1* and *TFDP1* (Supplementary Fig. 6a). These factors form heterodimeric complexes, which negatively impact immune activity^37^ and are directly involved in cell cycle progression; thus, *E2F1* is a potential dual-action regulatory target in this subgroup.

**Fig. 2:**
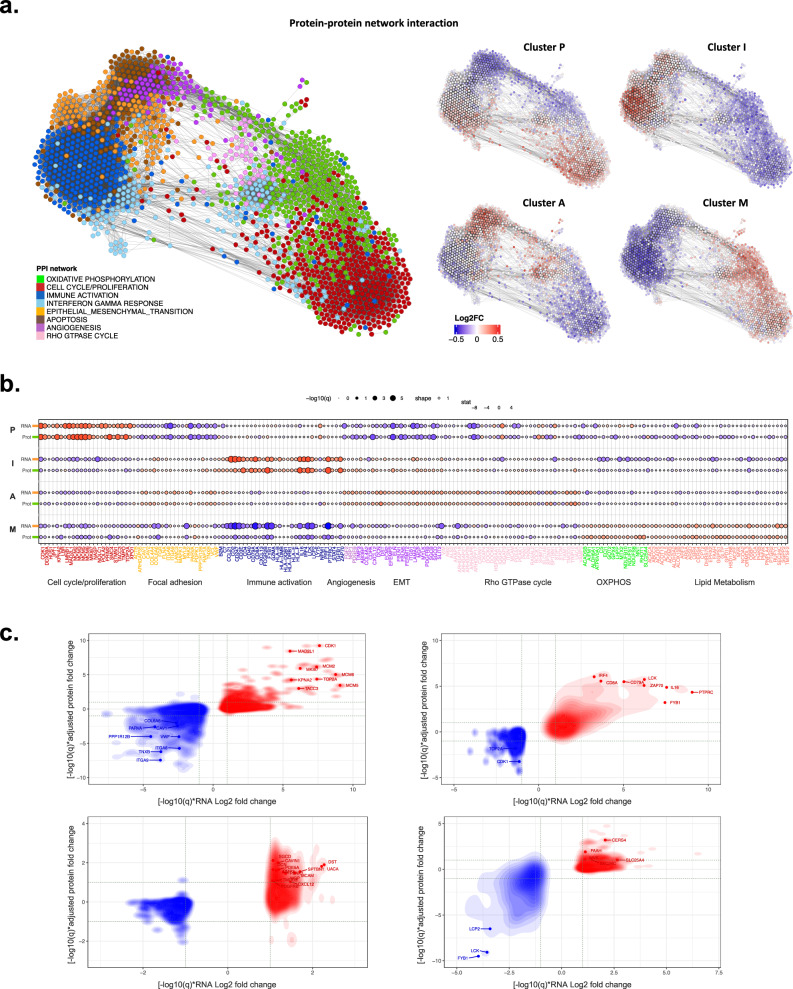
Proteome correlation clustering and characteristics associated with subgroups. **a** Protein correlation network of NSLA patients. Protein groups are defined and colored based on enrichment analysis of hallmark gene sets. Subtype-specific enrichment was also colored in red when it was higher and blue when it was lower than that in other subgroups. **b** Transcriptomic and proteomic expression of pathway markers depicting the characteristics of each subgroup. The size of the dot indicates significance. **c** Weighted rank density scatter plot indicating the magnitude of change multiplied by the significance of the protein expression on the y-axis and transcriptomic expression on the x-axis.

**Fig. 3:**
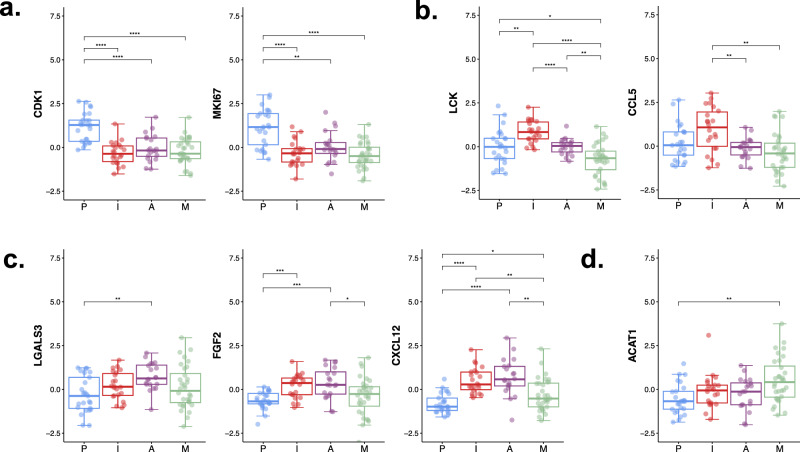
Candidate protein biomarkers highly expressed in each subgroup. Boxplots of candidate protein biomarkers: (**a**) CDK1 and MKI67 were overexpressed in the proliferation-high subgroup; (**b**) LCK and CCL5 were overexpressed in the immune-high subgroup; (**c**) the proangiogenic markers LGALS3, FGF2, and CXCL12 were overexpressed in the angiogenesis-high subgroup; (**d**) ACAT1 was overexpressed in the metabolism-high subgroup.

Proteome- and transcriptome-based enrichment in this subgroup revealed signaling networks centered on the cell cycle, chromosome modification, and DNA replication (Fig. 2a, b, Supplementary Figs. 8a, e, 9a, 10a). The weighted rank analysis of both the proteome and transcriptome in the P subgroup highlighted the upregulation of proliferation markers such as *CDK1*, *MAD2L1*, and the *MCM* family, while focal adhesion markers were downregulated (Fig. 2b, c). Analysis of kinases associated with the P subgroup identified numerous actionable targets, such as *CDK2* and *CDK5*, polo-like kinases (*PLKs*), and *ATR* (Supplementary Fig. 10a). Inhibition of *CDK2* can block hyperphosphorylation of the Rb protein, which leads to its binding to *E2Fs* and the *TFDP1* complex and blocking their target gene activation^38^. In clinical practice, elevated protein levels of *Ki67* and *CDK1* can be used as biomarkers for the identification of this subgroup (Fig. 3a).

### Unraveling immune dynamics and therapeutic implications in the immune-high (I) subgroup

Increased levels of antitumor immune components, such as T and B lymphocytes, major histocompatibility complex II (MHC II) pathway components, NK cells, and effector T cells, were observed in the I subgroup, with increased levels of protumor immune elements, such as regulatory T cells (Tregs), cancer-associated fibroblasts (CAFs), and immune checkpoint pathway molecules (Fig. 1a). B cells constituted a significant portion of the immune components, leading to a heightened presence within this subgroup (Supplementary Fig. 3f, *P* = 6.8e-9). The regulatory network exhibited a preference for beneficial immune activities, with upregulated immune-stimulating transcription factors such as *IRF4* and *TBX21* and downregulated immune-inhibitory regulators (Supplementary Fig. 6b). Notably, several immune-inhibitory transcription factors, including *E2F1*, were suppressed in this subgroup, suggesting an immune profile opposite to that of the P subgroup (Supplementary Fig. 6b). This subgroup also demonstrated a highly elevated representation of immune activation (Fig. 2a) and correlated enrichment of diverse immune-related signaling networks (Supplementary Fig. 8b, 8f, 9b). Furthermore, upregulation of immune activation marker genes and downregulation of oxidative phosphorylation marker genes were observed at both the RNA and protein levels, accompanied by the formation of a robust subnetwork of T-cell receptor (TCR) signaling pathways and high expression of the canonical T-cell signaling kinase *LCK* (Figs. 2b, 3b)^39^. Elements in the cytokine signaling pathway were upregulated at both the RNA and protein levels (Supplementary Fig. 9b, 10b). Several kinases that were significantly increased in the I subgroup constituted a network centered on immune cytokine signaling (Supplementary Fig. 10b), including *ZAP70* and *SYK*, which are known to regulate the maturation of T cells^39^.

**Fig. 4:**
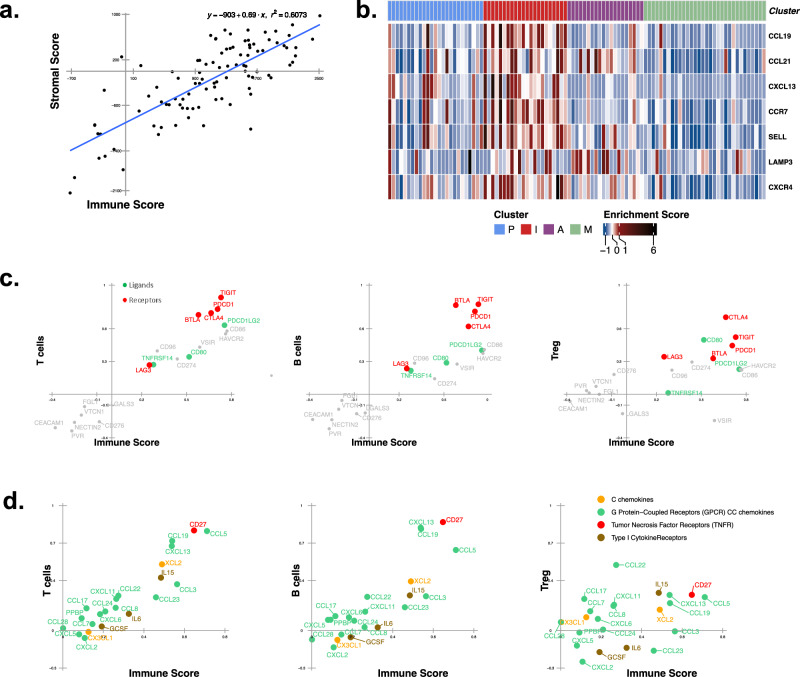
Immunological characteristics of the immune-high (I) subgroup. **a** Linear regression of immune and stromal scores depicting the concurrent increase in the stromal score and immune score (R^2^ = 0.61). **b** Heatmap representation of the expression patterns of tertiary lymphoid structure (TLS) marker genes. **c** Scatter plot for the correlation coefficient vectors between the immune checkpoint molecules and immune score calculated using the ESTIMATE algorithm as the x-axis and the RNA expression of the immune checkpoint molecules and T cell, B cell, and regulatory T cell scores as the y-axis. The immune checkpoint receptors are shown in red, the ligands are shown in green, and the expression of the molecules was determined using RNA-seq data. **d** Scatter plot of the correlation coefficients between cytokine and chemokine levels and the immune score. Type I cytokine receptors are shown in brown, G protein-coupled receptor (GPCR) CC chemokines are shown in green, C chemokines are shown in yellow, and tumor necrosis factor receptors (TNFRs) are shown in red.

Immune checkpoint inhibitors are the first-line treatment for metastatic NSCLC^40^ but are generally less effective in never-smokers than in smokers. The immunological reason for this reduced effectiveness in NSLA patients remains unclear. However, one hypothesis attributes it to a lower TMB and PD-L1 expression. To analyze the immunomodulatory mechanisms of this cohort, we performed a systemic evaluation of inhibitory receptors and their corresponding ligands (Fig. 4a–c, Supplementary Fig. 11a). The stromal and immune scores exhibited concurrent increases in various immune components in NSLA patients (Fig. 4a). Many samples exhibited B-cell enrichment and corresponding upregulation of tertiary lymphoid structure markers (Fig. 4b). Most inhibitory receptors, including *PD-1*, *TIGIT*, *BTLA*, and *CTLA4*, were upregulated in the I subgroup and were strongly correlated with immune cell infiltration^22^ (Fig. 4c). In contrast, *B7-H3*, *CEACAM1*, and *NECTIN2* were marginally upregulated in the nonimmune-high group and inversely correlated with T-cell infiltration (Supplementary Fig. 11a).

Effective immunotherapy requires adequate T-cell recruitment into tumors, a process in which cytokines and chemokines play crucial roles^41^. Both the proteome and transcriptome levels of immune activation markers, including TCR, were upregulated in the I subgroup, in addition to *CD8A, CD79A*, and *CD247* (Fig. 2b, c). The expression levels of cytokines and chemokines were strongly correlated with tumor immune scores and T-cell infiltration (Supplementary Fig. 12a). Among them, *CCL5* was differentially expressed at both the RNA and protein levels and showed the strongest positive correlation with the immune score (Figs. 3b, 4d, Supplementary Fig. 12a). In addition, *CXCL13* and *CD27*, which are cytokines that attract B cells and follicular helper T cells, were specifically elevated in the I subgroup (Supplementary Fig. 12a).

### Proteogenomic characteristics of the angiogenesis-high (A) subgroup

The A subgroup displayed certain similarities to the immune group in terms of a downregulated immune suppressive milieu; however, it did not exhibit an explicitly favorable immune regulatory network (Supplementary Fig. 6c), indicating the potential for enhancing immune activity through the utilization of immune activators (Supplementary Fig. 8c, 8g). Patients with the highest angiogenesis scores tended to be mutually exclusive with the P subgroup: 22/24 patients with the highest angiogenesis scores belonged to the nonproliferation-high subgroup (Fisher test, *P* = 0.018). Furthermore, samples with co-mutations in *KRAS* and *TP53* were found to be enriched in this subgroup. However, the characteristics of immune exclusion were apparent in the A subgroup, highlighted by the presence of CAFs and sporadically elevated levels of myeloid-derived suppressor cells (Fig. 1a), potentially impeding CD8 + T-cell infiltration^42^. The A subgroup demonstrated statistically significant enrichment of networks, including the *TGF-β*, cell migration, and angiogenic pathways (Supplementary Figs. 8c, 9c, 10c).

Various proangiogenic factors, including *FGF2* derived from CAFs^43^, *CXCL12*/SDF-1 secreted by human bone marrow stromal cells^44^, *PDGFB*, and pro-angiogenic *LGALS3*/galectin-3, were highly expressed at both the RNA and protein levels (Figs. 2b, c and 3c)^45^. When upregulated, *LGALS3* binds to integrin or *VEGFR2* on the endothelial cell surface, promoting the secretion of granulocyte colony-stimulating factor (G-CSF) and interleukin-6 (IL-6). Subsequently, IL-6 stimulates the NOTCH ligands *JAG1*/Jagged1 and DLL4, which play nuanced but distinct roles in angiogenesis^46^. Similarly, G-CSF derived from galectin-3 may also promote tumor growth by stimulating angiogenesis^47^.

### Proteogenomic enrichment analysis revealed lipid metabolism pathways in the metabolism (M) subgroup

The tumor samples from the M subgroup exhibited evident enrichment in oxidative phosphorylation, lipids, and carbon metabolism, along with other metabolic pathways (Fig. 2a, b, Supplementary Fig. 8d, 8h, 9d). Several upregulated kinases or related proteins in this subgroup, such as the *ERBB3*, *ICK*, *ARAF*, and CaMK2 families, were also associated with the MAPK cascade (Supplementary Fig. 10d), potentially affecting cholesterol homeostasis by promoting the expression of sterol regulatory element-binding proteins (SREBPs), which are transcription factors that regulate cholesterol synthesis^48^. This subgroup exhibited marginally immunosuppressive feature, and the expression of the acyl-CoA:cholesterol acyltransferase 1 protein (ACAT1), a suppressor of the proliferation of CD8 + T cells, was elevated (Fig. 3d, Supplementary Fig. 6d). The expression of acyl-CoA:cholesterol acyltransferase 1 protein (ACAT1), a suppressor of the proliferation of CD8 + T cells, was elevated in the M subgroup (Fig. 3d). The overexpression of ACAT1 may be involved in depleting the cholesterol required for TCR clustering in CD8 + T cells and decreasing T-cell avidity to antigens by esterifying free cholesterol. Moreover, the expression of CERS4, a pivotal enzyme in sphingolipid metabolism, was upregulated at both the RNA and proteome levels (Fig. 2b, c). Prior research has established a positive correlation between CERS4 and the efficacy of anti-PD-1 therapy in NSCLC^49^.

**Fig. 5:**
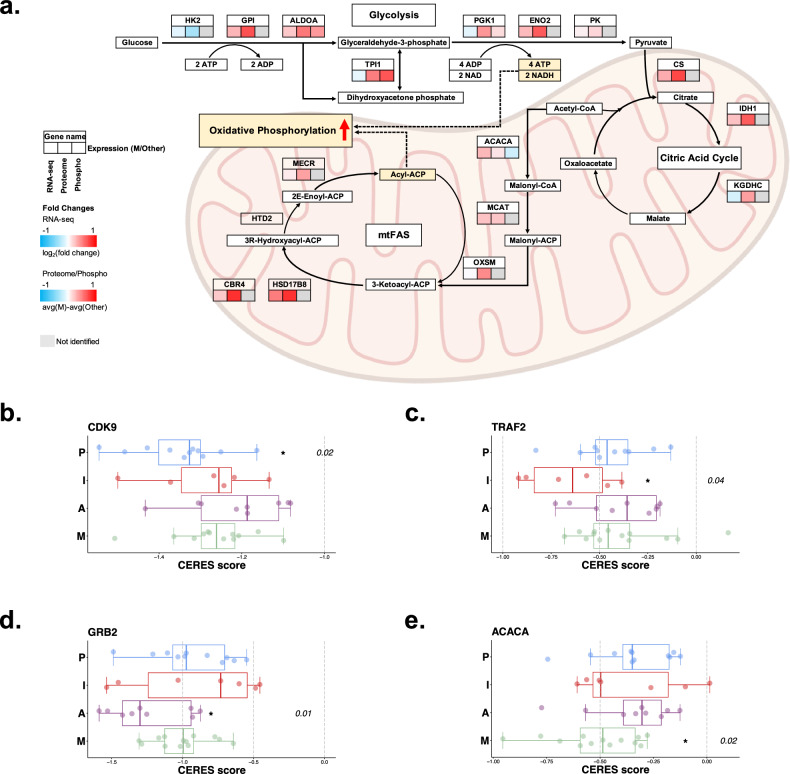
Metabolomic subgroups with upregulated pathways and subtype-specific vulnerabilities. **a** Signaling pathway diagram of glycolysis, the citric acid cycle, and oxidative phosphorylation showing significantly upregulated expression in the M subgroup. **b** Boxplots showing a significantly greater dependency on the CDK9 gene observed exclusively within the P subgroup (*P* = 0.02). **c** The TRAF2 gene exhibited heightened dependency on the I subgroup (P = 0.04), (**d**) the GRB2 to A subgroup (*P* = 0.01), and (**e**) the ACACA to M subgroup (*P* = 0.01).

Our proteogenomic data revealed coordinated enhancement of the citric acid cycle, the pentose phosphate pathway, and nucleotide biosynthesis, which, in turn, influenced amino acid and lipid metabolism (Fig. 5a, Supplementary Fig. 8h). Transcriptomic and proteomic levels of acetyl-CoA carboxylase alpha (*ACACA*), which catalyzes the conversion of acetyl-CoA to malonyl-CoA for mitochondrial fatty acid synthesis (mtFAS), were increased in the M subgroup^50^ (Fig. 5a).

### Subtype-specific cancer vulnerabilities

We analyzed data from the CCLE^51^ and DepMap^29^ databases to identify vulnerabilities and potential treatment avenues in the NSLA subgroups. Our initial analysis specifically focused on 69 lung adenocarcinoma (LUAD) cell lines without oncogenic *EGFR*/*ALK* mutations. Pathway enrichment was conducted using marker gene lists, and cell lines were subsequently grouped into four distinct subgroups using the same algorithm applied to the NLSA cohort (Supplementary Fig. 13a)^37^. The similarity of the four clusters between the bulk KNCC cohort and cell line cohort was analyzed with Spearman correlation, which revealed a strong correlation between the same subtype in the two different cohorts (Supplementary Fig. 13b). To identify subgroup-specific targets, we delved into genes exhibiting a significantly substantial impact upon subgroup perturbation. Our findings revealed that the P subgroup exhibited a greater dependency on the *CDK9* gene than did the other subgroups (*P* < 0.05, Fig. 5b).

The cell lines in the I subgroup showed a strong dependency on *TRAF2* (Fig. 5c). The presence of tumor cell-expressed *TRAF2* has previously been recognized as a significant factor that restricts the ability of cytotoxic T cells to eliminate cancer cells even after immune checkpoint blockade^52^. The A subgroup demonstrated a pronounced dependency on *GRB2*, which is intricately linked to the EGFR pathway (Fig. 5d). The cell lines belonging to the M subgroup exhibited notable reliance on cancer metabolic genes such as *ACACA*, the pivotal player of mtFAS (Fig. 5e). Collectively, our results underscore the distinct gene dependencies of the P, I, A, and M subgroups on *CDK9*, *TRAF2*, *GRB2*, and *ACACA*, respectively.

The PRISM Repurposing dataset was utilized to assess drug sensitivity in lung cancer cells across each subgroup, revealing several drug matches, including digitoxin and tacedinaline, as promising compounds for inducing cancer cell death in the P subgroup (Supplementary Fig. 13c). KI16425, a lysophosphatidic acid receptor (LPAR) antagonist, is suggested for treating subgroup I patients (Supplementary Fig. 13d). The I subgroup exhibited significantly greater *LPAR6* expression than the other subgroups (Supplementary Table 10, *P* = 0.002), which was previously associated with negative regulation of CD8 + T-cell tumor infiltration^53^. For the A subgroup, ibutamoren (MK-677), which is a synthetic compound and a growth hormone secretagogue that mimics the action of ghrelin by binding to the ghrelin receptor and increasing the release of growth hormone, was selected (Supplementary Fig. 13e). Ghrelin was previously shown to protect against hypoxia-induced lung injury by preventing hypoxia-induced increases in angiogenesis and HIF1-alpha and *VEGF* expression^54^. Finally, clorsulon was suggested for the M subgroup (Supplementary Fig. 13f). Clorsulon is widely used as an anthelmintic in calves and sheep but is also a competitive inhibitor of both 3-phosphoglycerate and ATP, inhibiting glucose utilization and acetate and propionate formation^55^.

### Identification of cancer-specific antigens

**Fig. 6:**
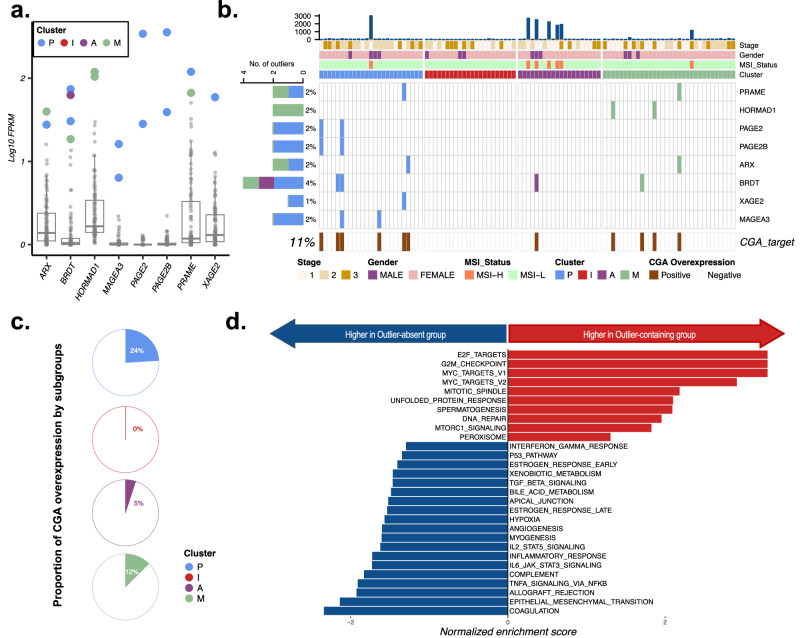
Cancer-specific antigens in NSLA. **a** Boxplots of cancer-specific antigen expression, log-scaled at the transcriptomic level on the y-axis, depicting outlier expression by different colors belonging to each molecular subtype of NSLA. **b** Heatmap illustrating the distribution of samples with cancer-specific antigen expression. **c** Pie graph showing the proportion of CGA-positive samples by subgroup. The subgroup with the highest percentage of samples exhibiting CGA overexpression was the P subgroup (28%), followed by the M subgroup (22%), the A subgroup (5%), and the I subgroup, which did not display marked overexpression of any CGAs. **d** Pathway enrichment analysis using hallmark gene sets from MSigDB associated with tumors expressing cancer-specific antigens.

Cancer germline antigens (CGAs), which are exclusively present in normal germ cells and some cancer cells, are considered promising targets for cancer immunotherapy due to their potential to enhance treatment efficacy while minimizing patient toxicity^30^. To identify patients who are most likely to benefit from targeting cancer-specific proteins, we selected CGA lists with at least one outlier whose expression was a minimum of 100-fold greater than the average expression (Fig. 6a). In our cohort, 11% of the samples exhibited atypically elevated CGA expression and were mostly clustered in nonimmune subgroups (Fig. 6b). The prevalence of CGA overexpression varied among the subtypes (Fig. 6c). The P subgroup had the greatest proportion of CGA-overexpressing samples (24%), while the I subgroup did not exhibit any instances of CGA overexpression, with the deficiency of CGA overexpression in the A and I subgroups (Fisher test, *P* = 0.02). To gain deeper insights into the molecular distinctions associated with the overexpression of CGAs, we conducted a differentially expressed gene analysis between patients with and without CGA expression (Supplementary Fig. 14c, Supplementary Table 12). The downregulated genes in the CGA-containing group were mainly related to immune-related genes and pathways, such as interleukin family signaling and the interferon response (Fig. 6d). The same analysis was conducted with the never-smoker subgroup of the TCGA-LUAD cohort (n = 46). Although statistical significance was not detected due to the limited sample size (*P* = 0.18), a trend toward underrepresentation of CGA-positive cancers in subgroups A and I was observed, and immune-related genes were downregulated in CGA-positive samples, similar to our current cohort (Supplementary Fig. 14a–d). To compare the increased expression of CGA markers within our patient cohort relative to that in normal tissue, we conducted a comparative analysis using normal tissue data sourced from the GTEx project^31^, revealing significantly elevated expression levels of specific CGAs exclusively within cancer and germline tissues in individual patients (Supplementary Fig. 15).

## Discussion

Our comprehensive proteogenomic analyses revealed a nuanced understanding of the tumor microenvironment in NSLA patients devoid of oncogenic *EGFR* and *ALK* alterations. Contrary to the expectations of a homogeneous group, our findings revealed a diverse array of characteristics within this cohort, reflecting the complex nature of the disease. The absence of *EGFR* driver mutations or *ALK* rearrangements does not equate to a uniform pathology; instead, it highlights the multifaceted nature of the cohort. This diversity encompasses variations in proliferation rates, angiogenic potential, the immune landscape, and metabolic profiles, emphasizing the need for tailored therapeutic approaches. Understanding these subtypes is crucial for devising effective treatments. The P subgroup, characterized by elevated proliferation rates and *TP53* mutations, may resist apoptosis-inducing drugs. Targeting *CDK*s, *PLK*s, *ATR*, and *E2F1*, in addition to increasing DNA damage repair activity, may offer therapeutic opportunities for this subgroup^37^.

Our analysis indicated that the I subgroup is highly receptive to immunotherapy due to elevated levels of immune components and related signaling networks. Increased expression of inhibitory receptors and their ligands may contribute to the reduced effectiveness of ICIs in NSLA patients. Variable expression of cytokines and chemokines, notably *CCL5*, *CXCL13*, and *CD27*, suggests potential targets for combination immunotherapy within this subgroup^56,57^. *CCL5* expression is induced during the later stages of inflammation, during which activated effector T cells, NK cells, M1 macrophages, and memory T cells are recruited to the inflamed area. Additionally, *CCL5* specifically recruits Tregs, which can suppress antitumor immunity or directly facilitate metastasis. Furthermore, *CXCL13* expression was highly upregulated in the I subgroup, attracting B cells and follicular helper T cells^56^. The presence of B cells and tertiary lymphoid structure markers in certain samples underscores the significance of B-cell activity^57^. A recent study showed that a lack of *CXCL13* enhanced the efficacy of anti-PD-1 immunotherapy^57^. Additionally, *CD27* upregulation in this subgroup coincides with that of *TRAF2*, the canonical adapter of *CD27*, which was identified as the top hit in a genome-wide CRISPR screen for genes that sensitize tumor cells to T-cell-mediated elimination when *CD27* is knocked out^58^. These findings reveal a complex interaction between antitumor and protumor immune components and highlight the potential utility of personalized immunotherapy for NSLA patients.

The A subgroup has a high prevalence of angiogenesis characteristics, suggesting potential avenues for angiogenesis-related therapeutic interventions. This subgroup included the most patients with the *KRAS* G12V genotype (Supplementary Table 7). The *KRAS* G12C mutation is normally the most common mutation type in lung adenocarcinoma patients (up to 45%) and is strongly associated with a poor prognosis and smoking status^59^. Consistent with the nature of the current cohort, which comprised patients who were lifelong never smokers, the G12C mutation type was found in a minor portion of patients in the NSLA cohort. This subgroup’s frequent *TP53* and *KRAS* gene mutations underline the considerable influence exerted by these mutations on both the extracellular environment and intracellular processes. A previous study showed that concurrent *KRAS* and *TP53* mutations in lung cancer are accompanied by high CD8 + T-cell infiltration and *PD-L1* levels, suggesting that these patients are candidates for αPD-L1 immune checkpoint blockade^60^. The overexpression of *LGALS3*, *CXCL12*/SDF-1, and *FGF2* in this subgroup suggests these factors as prospective therapeutic targets^43–46^. Specifically, inhibiting *LGALS3* enhances the secretion of G-CSF and IL-6 by binding to integrin or hypoxia-induced *VEGF* and is a promising therapeutic approach for this subgroup^46,54^.

Our clustering analysis offers valuable insights into molecular subtypes, yet the limitations of bulk-level transcriptome and proteome data exist in discerning signals from surrounding tumor microenvironment cells versus cancer cells. While immune and angiogenic features could originate from tumor microenvironment cells, introducing heterogeneity, we were able to cluster CCLE cell lines with similar features, albeit less pronounced than in the bulk data. However, future studies should incorporate single-cell analysis to discern the origin of these features more unambiguously.

An increasing number of studies are exploring the potential of CGAs as promising candidates for cancer vaccines or targets for adaptive T-cell therapy^30^. The CGAs in this study were extensively studied for their pseudomeiotic functions, facilitating alternative DNA repair mechanisms linked to cancer progression and therapeutic resistance. These CGAs, except *ARX*, are typically not expressed in normal female cells, offering an advantage in our mostly female cohort (86.9%). Targeting these CGAs in never-smoker women with lung cancer may lead to reduced side effects. This finding suggests the potential for immune-activating strategies targeting these antigens in preselected NSLA patient subsets.

Our analysis identified 4 NSLA subtypes via proteogenomic profiling, offering distinct insights into the tumor microenvironment. The prospective application of proteogenomics in real biopsy samples has the potential to refine treatment strategies for distinct molecular subtypes identified through clustering analysis, offering insights into the underlying mechanisms, guiding the selection of targeted therapies tailored to each subtype’s specific characteristics and providing enhanced prognostic power. These subtypes revealed unique biological mechanisms and therapeutic targets that can be used to guide personalized treatment for NSLA patients lacking *EGFR* and *ALK* mutations. We characterized each subtype’s molecular features and recommended treatments, highlighting subtype-specific therapeutic strategies.

## Supplementary information

Supplementary Information

Supplementary Table 1-12

## Data Availability

For the KNCC NSLA dataset, the raw genomic, transcriptomic, and epigenomic data were deposited in the Korean Nucleotide Archive (https://kobic.re.kr/kona) under accession IDs KAD2100075 and KAD2100133. All genomic and transcriptomic data are available from KoNA upon reasonable request. Processed datasets are provided in Supplementary Tables 1–3. All proteomic and phosphoproteomic data, including raw files, protein databases, and search results, were deposited in the Korea BioData Station (K-BDS) with the bioproject identifier PRJKA108675 and in the ProteomeXchange Consortium via the PRIDE partner repository with the dataset identifier PXD033360. The TCGA dataset was downloaded from the Genomic Data Common (GDC) data portal (https://portal.gdc.cancer.gov/) operated by the National Cancer Institute (NCI). The raw genomic, transcriptomic, and epigenomic data were deposited in the Korean Nucleotide Archive (https://kobic.re.kr/bps/kona) under accession ID PRJKA210029. All genomic and transcriptomic data for our cohort are available from the KoNA upon request. All proteomic data, including the raw files, protein databases, and search results, were deposited in the ProteomeXchange Consortium via the PRIDE partner repository with the dataset identifier PXD033360.
