# Supplementary material for: Proteogenomic characterization identifies clinical subgroups in EGFR and ALK wild-type never-smoker lung adenocarcinoma
Source: Exp Mol Med. 2024 Sep 19;56(9):2082–95. doi: 10.1038/s12276-024-01320-0 (PMC11446976; doi:10.1038/s12276-024-01320-0)
Supplement: Supplementary file 1 — Supplementary Information [file 12276_2024_1320_MOESM1_ESM.pdf]

Supplementary Figure 1

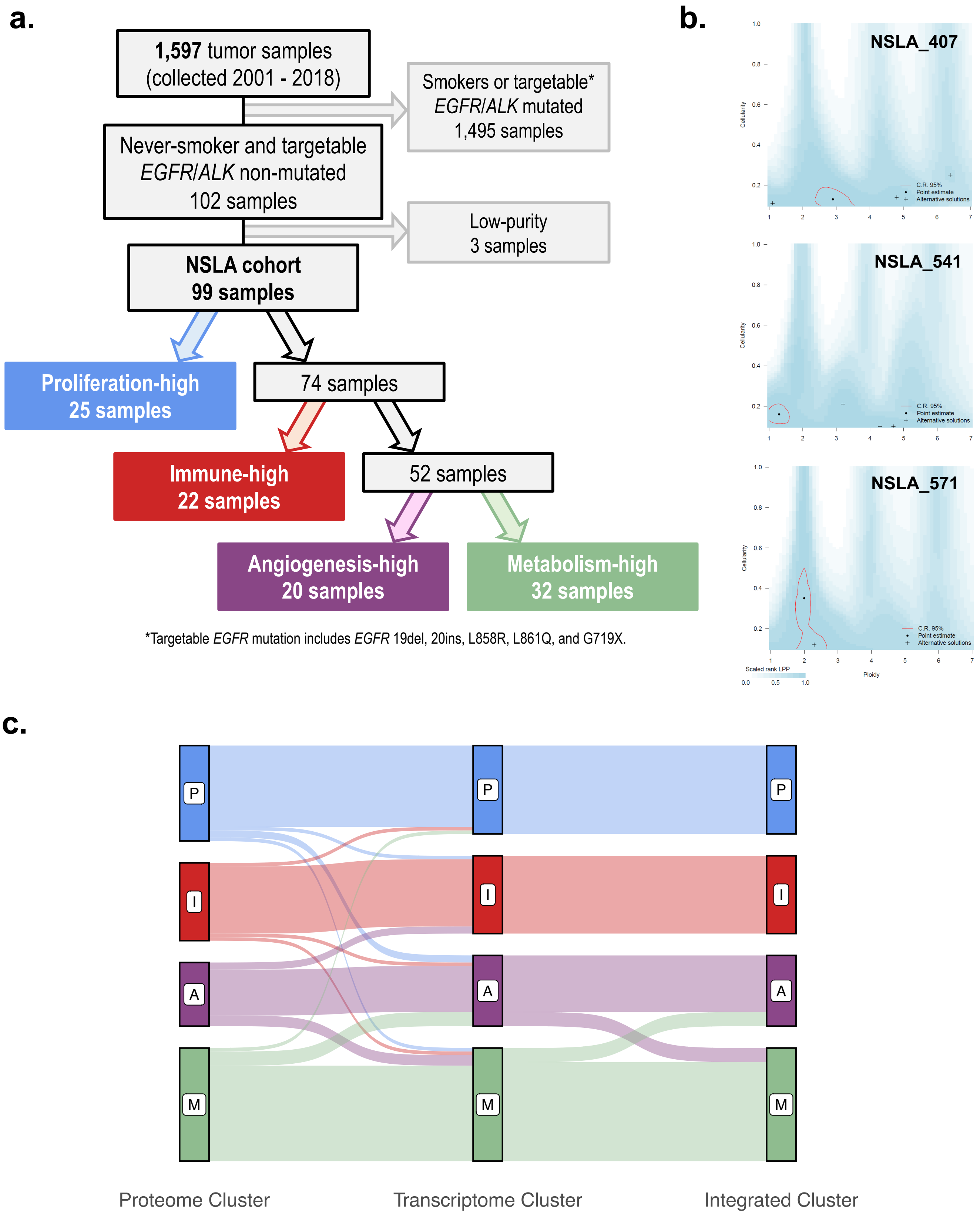

Supplementary Figure 1. Sample selection and classification into four distinct subgroups. **(a)** Schematic diagram of sample selection and classification of subgroups. Proliferation-high subgroup (n = 25) was first segregated followed by immune-high subgroup (n = 22), angiogenesis-high subgroup (n = 20) and metabolism-high subgroup (n = 32). **(b)** The 3 tumor samples were removed with low genomic purity verified by Sequenza algorithm using paired tumor and normal WGS data. Optimal purity, ploidy information is depicted as a block dot in the figure. **(c)** Sankey diagram visualizing distribution of transcriptome clustering, proteome clustering and integrated clustering.

Supplementary Figure 2

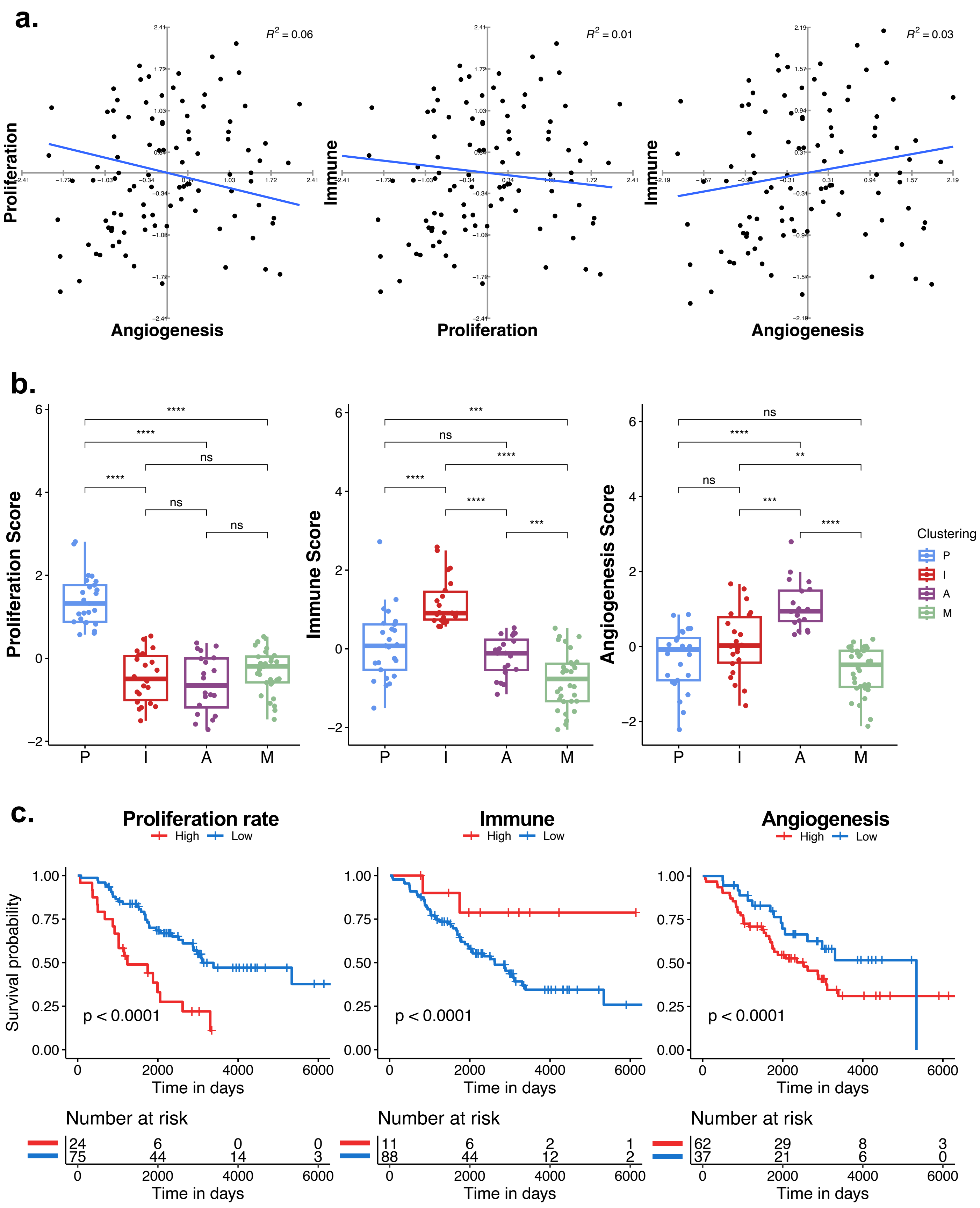

Supplementary Figure 2. Clinical association of three molecular factors used in the classification and determination of their interdependence

(a) Orthogonal indepenence between molecular gene signatures employed in the classification. The R-squared value was 0.009 between Proliferation and Immune, 0.0566 between Proliferation and Angiogenesis, and 0.0982 between Angiogenesis and Immune. (b) Boxplots of classification factors. The P, I, and A subgroups significantly differed from all other groups in proliferation rate, T cell signature score, and angiogenesis, respectively. (c) Kaplan-Meier plot of overall survival for segregated by proliferation, angiogenesis, and immune gene scores.

Supplementary Figure 3

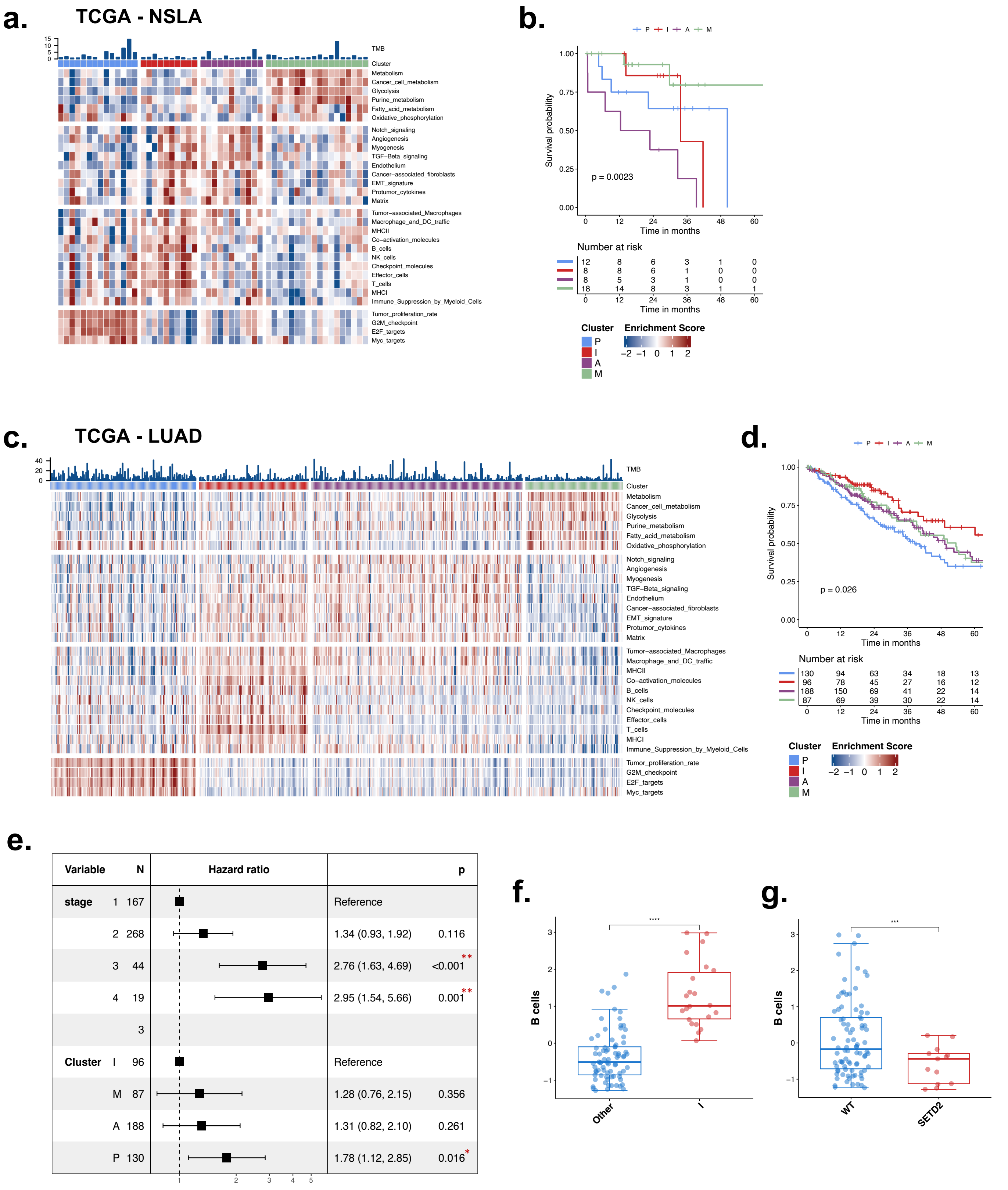

Supplementary Figure 3. Heatmap representation of 30 gene set signatures in TCGA lung adenocarcinoma data.

(a) The thirty TME scores were calculated using single sample gene set enrichment analysis of data from lung adenocarcinomas from TCGA excluding current/former smokers and *EGFR*/*ALK* mutated samples (TCGA-NSLA, n=46). **(b)** Overall survival probability of TCGA-NSLA cohort for validation of clinical association with molecular subtyping algorithm. **(c)** The thirty TME scores were calculated using single sample gene set enrichment analysis of data from 506 lung adenocarcinomas from TCGA, **(d)** Overall survival probability of The Cancer Genome Atlas (TGCA) lung adenocarcinoma cohort for validation of clinical association with molecular subtyping algorithm. **(e)** Forest plot for Cox proportional hazards model of TCGA-LUAD cohort. Multivariate analysis was conducted adjusting for gender, age, stage and subtype. **(f)** B lymphocyte gene set scores of immune-high subgroup and others and **(g)** those of mutant and wild type SETD2.

Supplementary Figure 4

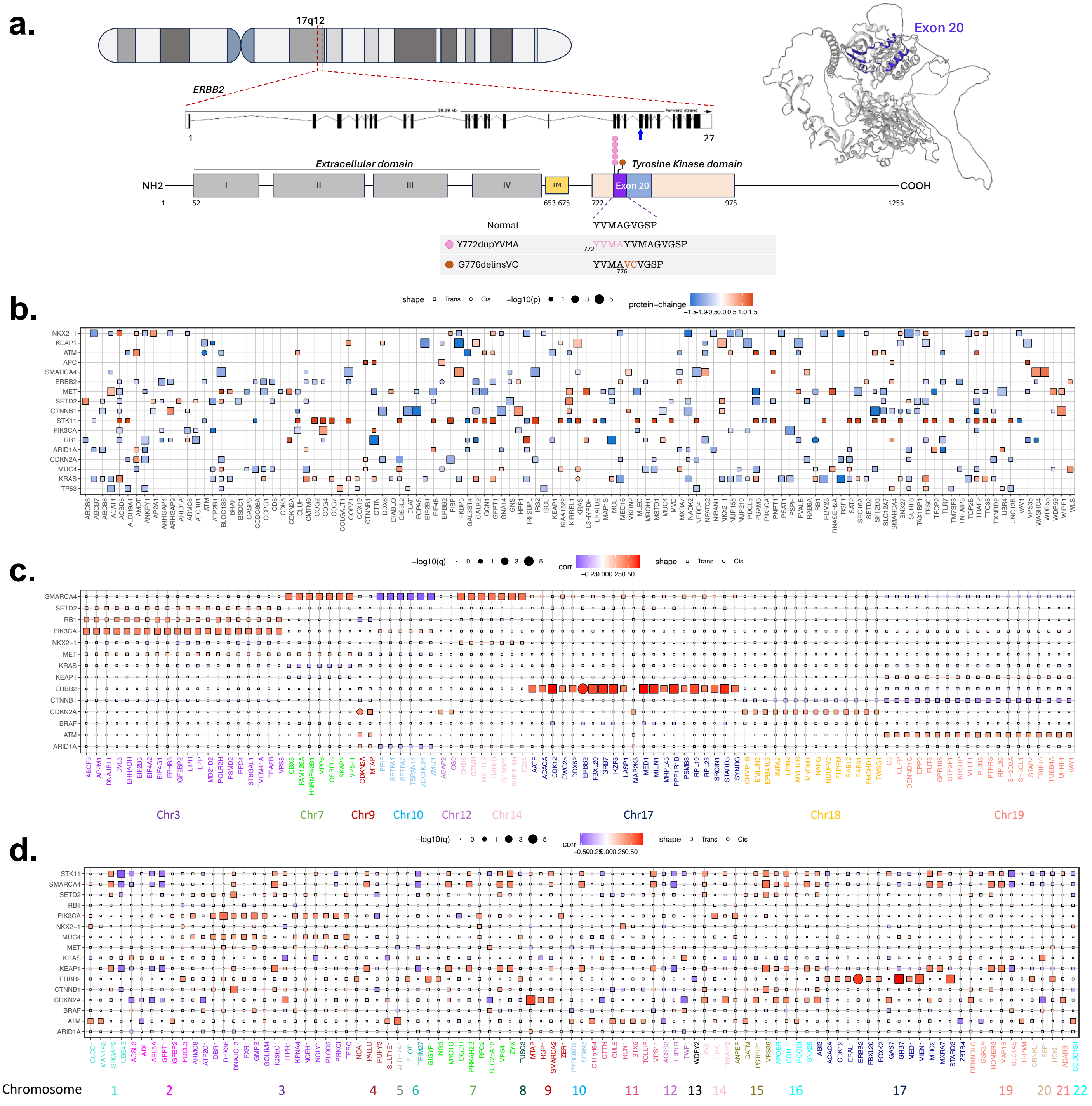

Supplementary Figure 4. impact of genetic alteration on proteogenomic landscape of NSLA cohort.

(a) Schematic description of ERBB2 chromosomal location exon20 insertion in NSLA cohort with 3D structure of exon 20 location (colored in blue). **(b)** The list of proteins (x-axis) that are influenced significantly by both cis- and trans-effects of somatic driver mutations (y-axis) with the threshold of FDR < 0.1. **(c)** The correlation between copy number alteration (x-axis) and protein abundance (y-axis) that are frequently mutated in NSLA cohort. **(d)** The correlation between protein abundance (x-axis) and copy number alteration (y-axis) that are frequently mutated in NSLA cohort

Supplementary Figure 5

a.

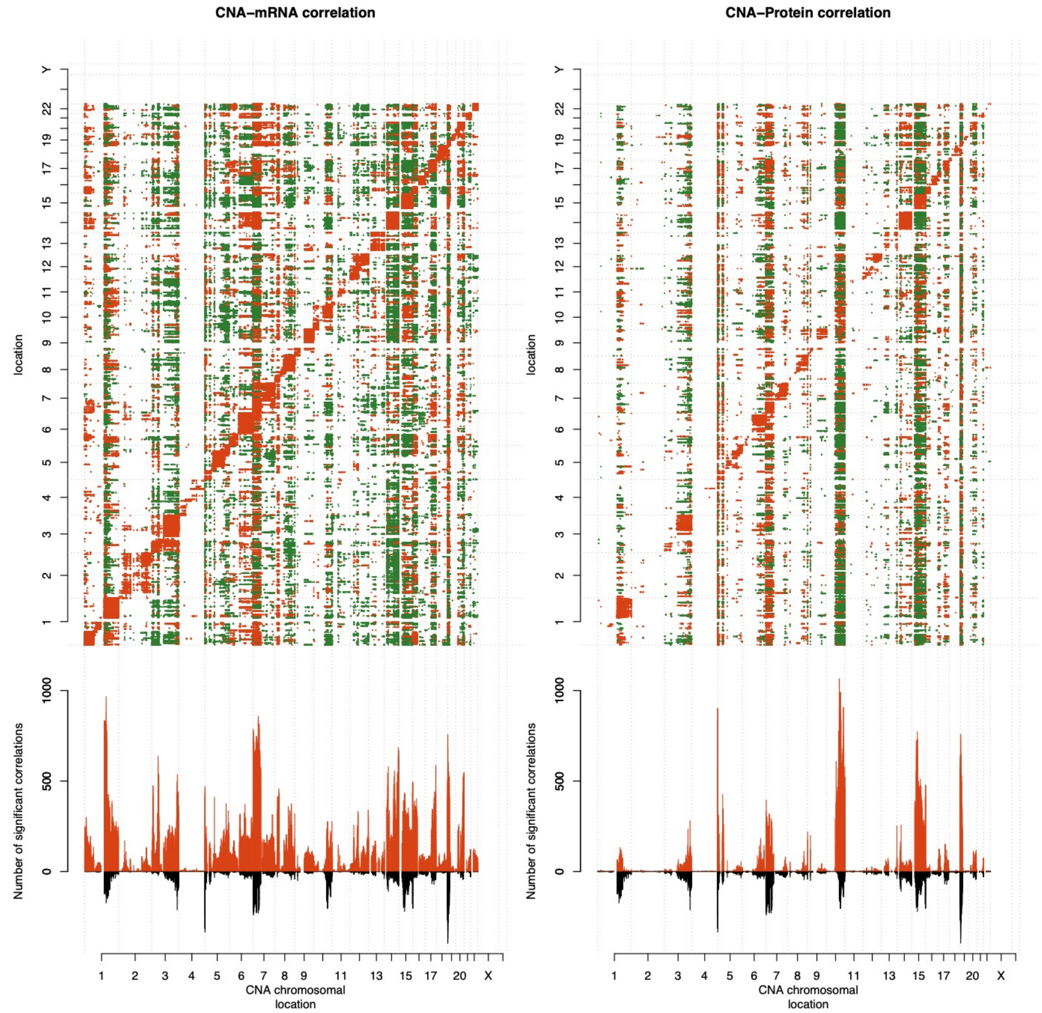

b.

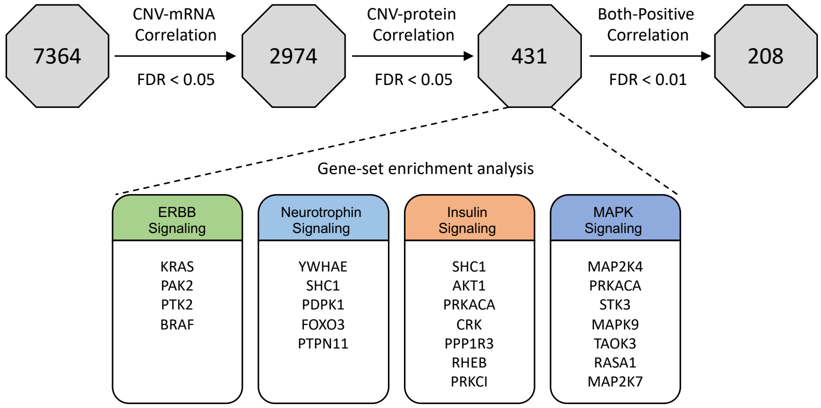

c.

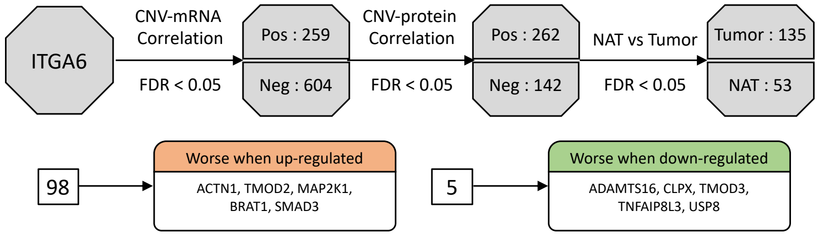

Supplementary Figure 5. Correlation between CNA, mRNA and Proteome, and pathway analysis of cis- and trans-acting genes

(a) The correlation graphs illustrate the association between CNA and both mRNA and protein abundance. Significant correlations are visually highlighted: positive correlations are depicted in red, while negative correlations are in green (FDR < 0.05). CNA-driven cis effects manifest as red diagonal lines, whereas trans effects are represented by vertical red and green lines. Accompanying histograms complement the graphs, providing insights into the frequency of significant cis and trans events at specific genomic loci. Separate histograms delineate the common correlation between CNA-RNA and CNA-protein events, depicted in black. Instances where correlation events are unique to either mRNA or protein are indicated by the color red. **(b)** Gene-set enrichment analysis of 431 filtered gene with threshold of correlation significance FDR < 0.05. **(c)** Trans-acting gene lists exerting significant influence on a wide range of other genes and with a notable impact on the overall survival of the associated proteins.

Supplementary Figure 6

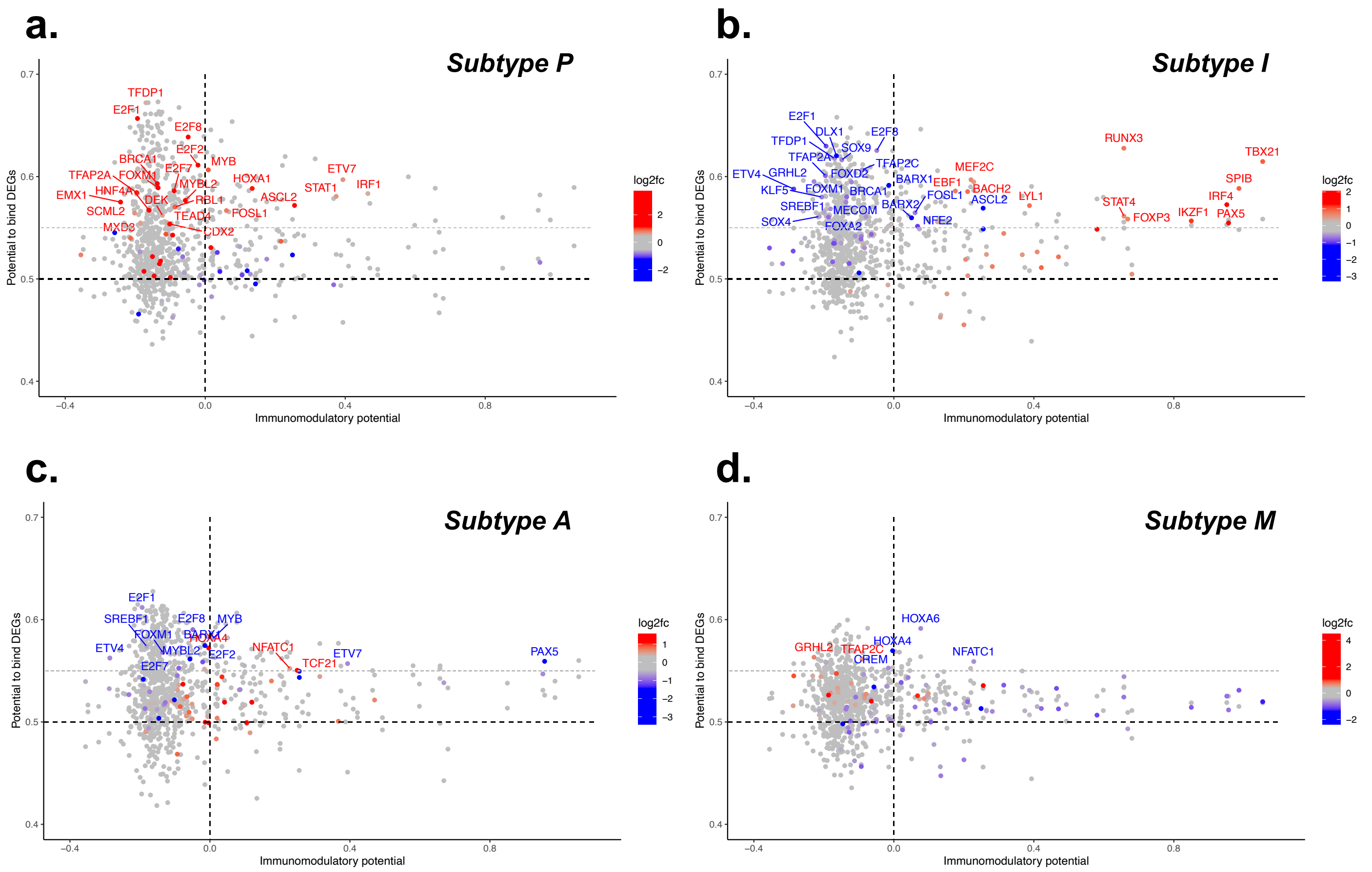

Supplementary Figure 6. Bipotent transcription factors or chromatin regulators (TFCRs) represented by differentially expressed genes with enriched pathway analysis from PPI networks for each subgroup

Discovered immunomodulatory potential for DEG and **(a)** 674 enriched genes in the proliferation-high (P) subgroup, **(b)** 489 enriched genes in the immune-high (I) subgroup, **(c)** 270 enriched genes in the angiogenesis-high (A) subgroup, and **(d)** 323 enriched genes in the metabolism (M) subgroup.

Supplementary Figure 7

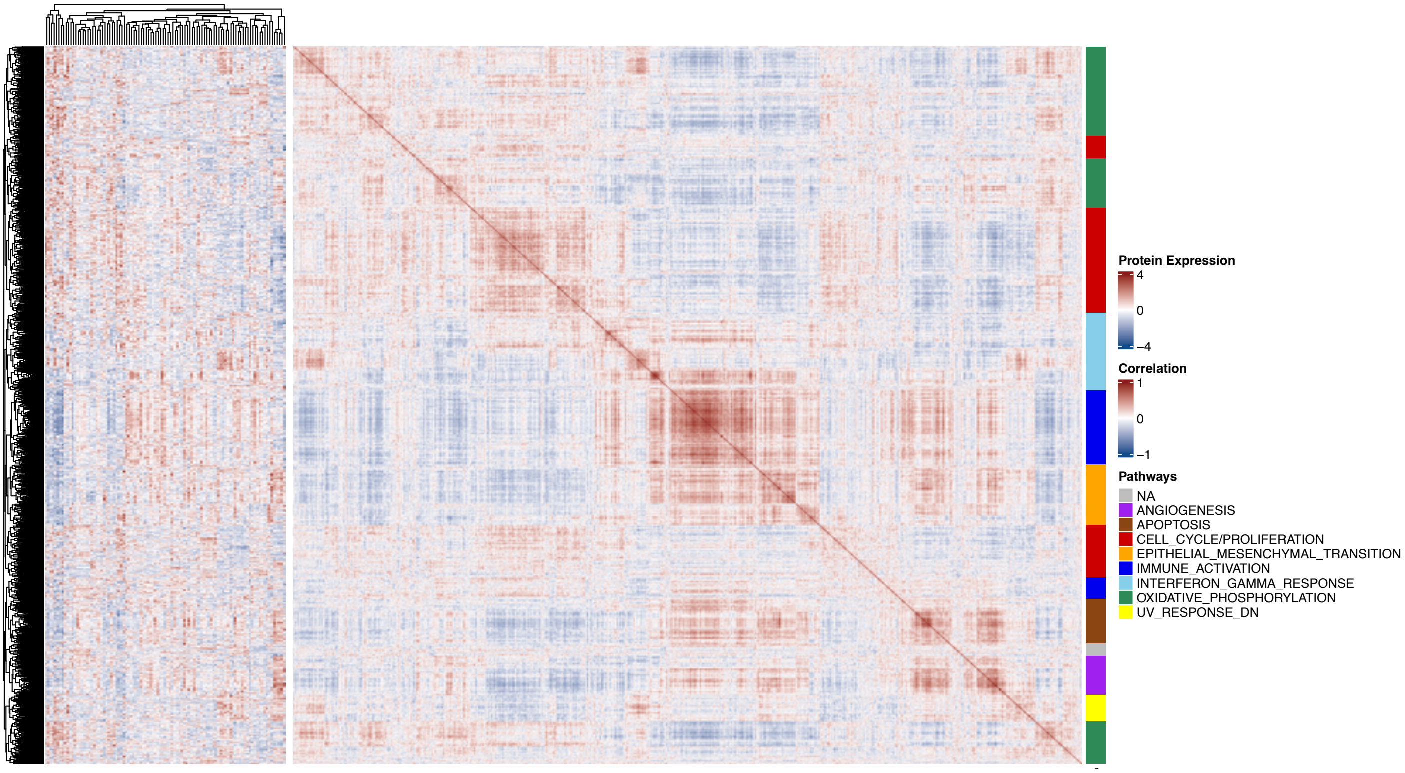

Supplementary Figure 7. Protein-protein correlation and associated molecular functions. Unsupervised hierarchical clustering ($P > 0.5$, complete linkage) performed on a protein correlation matrix comprising 2,063 high-variance proteins across a set of 99 samples. Associated pathways enrichment was analyzed using Hallmark gene sets from the Molecular Signature Database (MSigDB).

Supplementary Figure 8

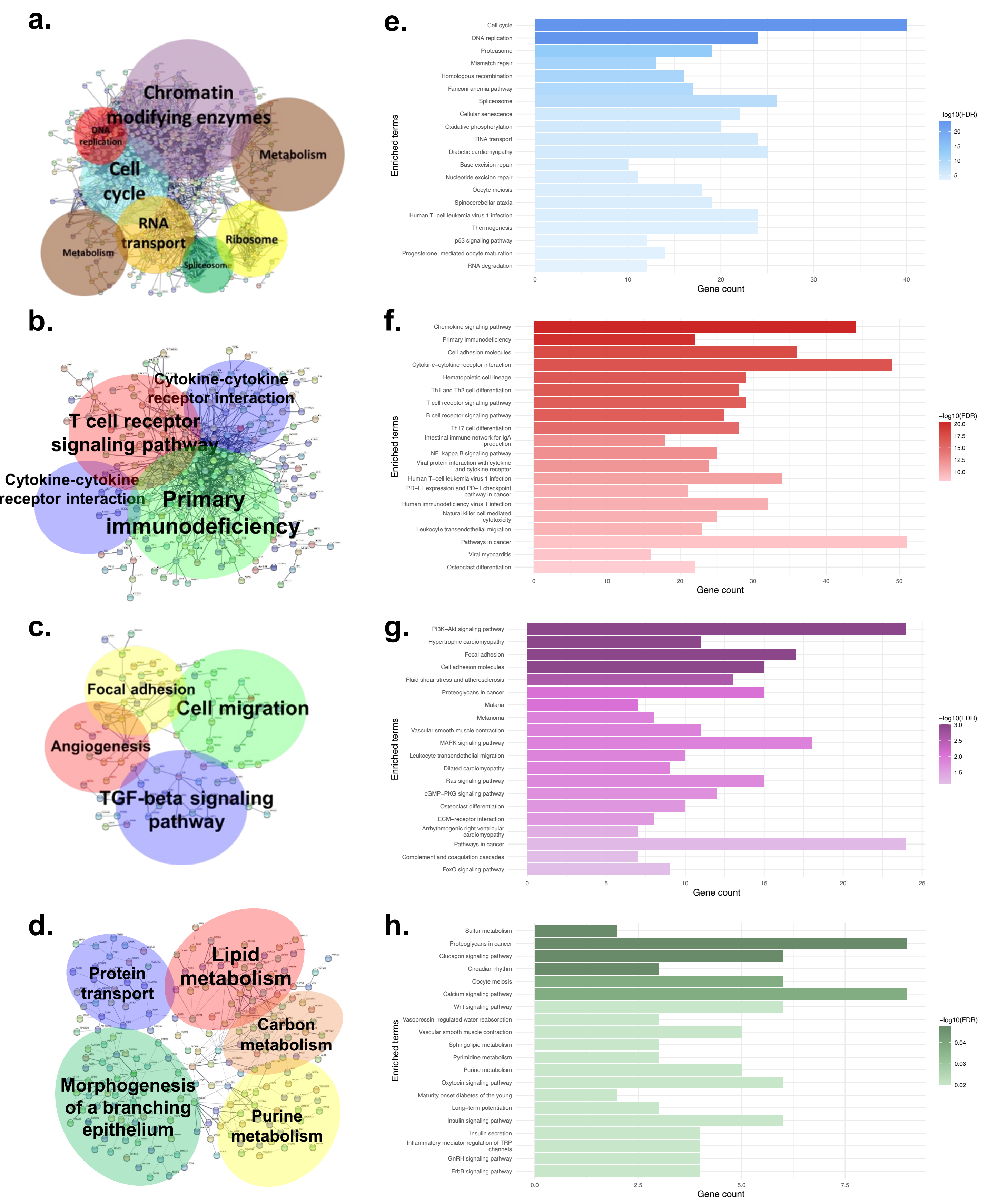

Supplementary Figure 8. Subtype-specific protein-protein interaction (PPI) networks and enriched pathway analysis.

PPI networks and bar plots illustrating the pathway enrichment results for different subgroups: **(a, e)** P subgroup with 674 enriched genes, **(b, f)** I subgroup with 489 enriched genes, **(c, g)** A subgroup with 270 enriched genes, and **(d, h)** M subgroup with 323 enriched genes. The PPI networks were generated using STRING (https://string-db.org/), and the bar plots depict statistically enriched gene set terms sourced from the KEGG_2023_Human database.

Supplementary Figure 9

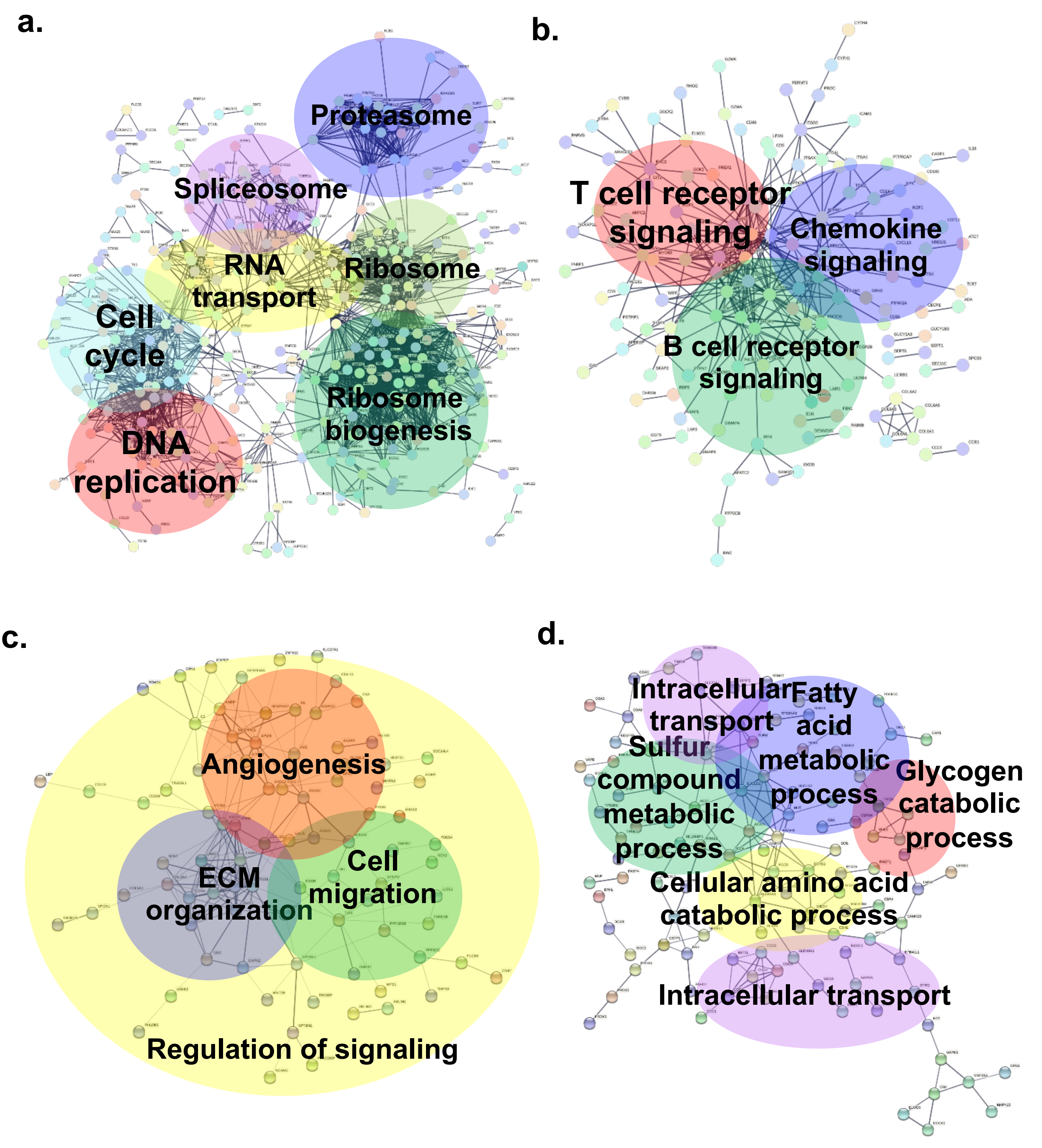

Supplementary Figure 9. Protein-protein interaction (PPI) networks based on proteome data.

PPI networks of **(a)** 437 enriched proteins in the P subgroup, **(b)** 333 enriched proteins in the I subgroup, **(c)** 120 enriched proteins in the A subgroup, and **(d)** 120 enriched proteins in the M subgroup.

Supplementary Figure 10

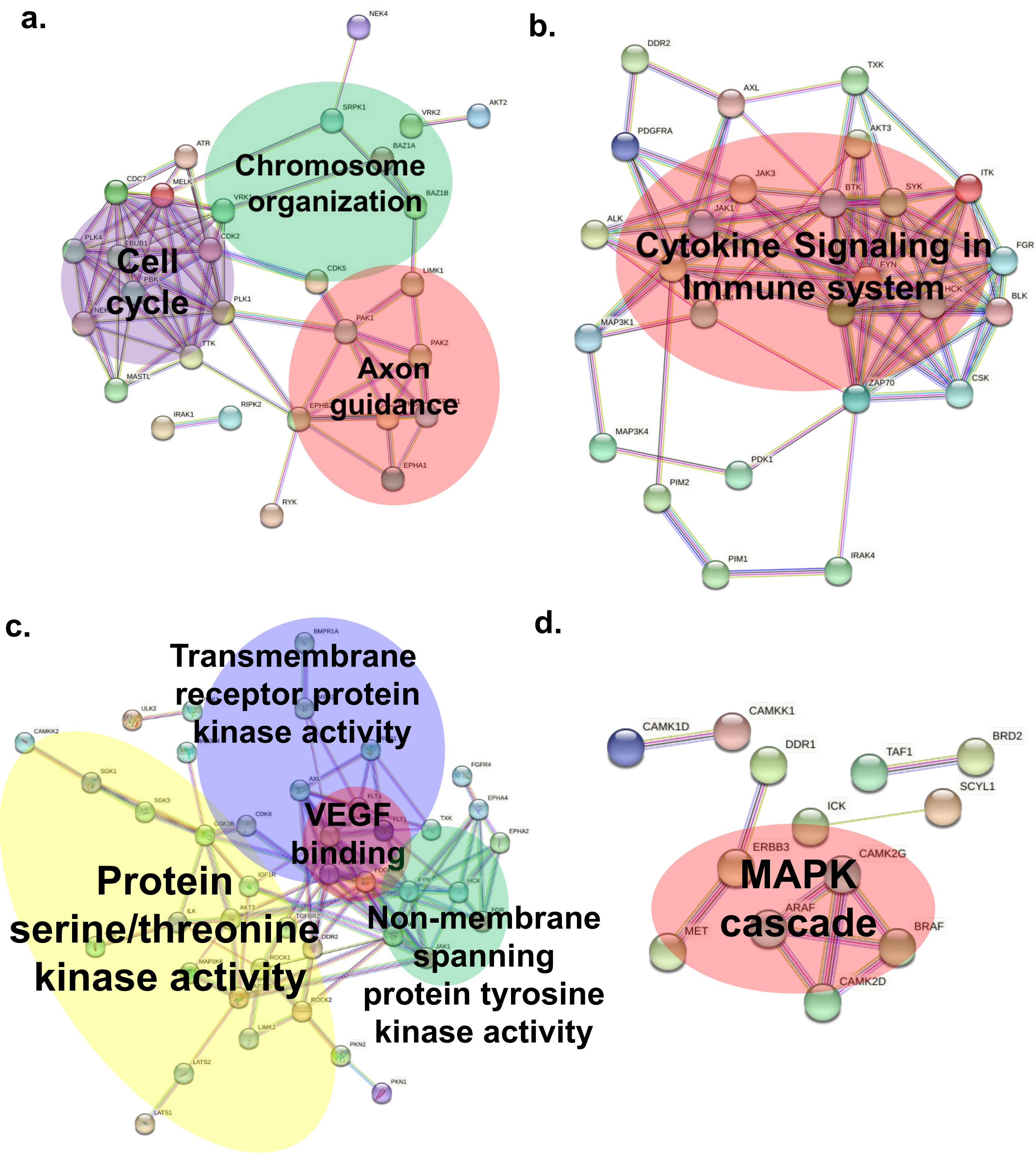

Supplementary Figure 10. Protein kinase interaction networks of each subgroup

These PPI networks were drawn involving kinases statistically significantly enriched in the subgroups. Nodes without any interactions are not shown. **(a)** PPI network of 34 kinases enriched in the P subgroup, **(b)** PPI network of 38 kinases enriched in the I subgroup, **(c)** PPI network of 62 kinases enriched in A subgroup, **(d)** PPI network of 32 kinases enriched in the M subgroup.

Supplementary Figure 11

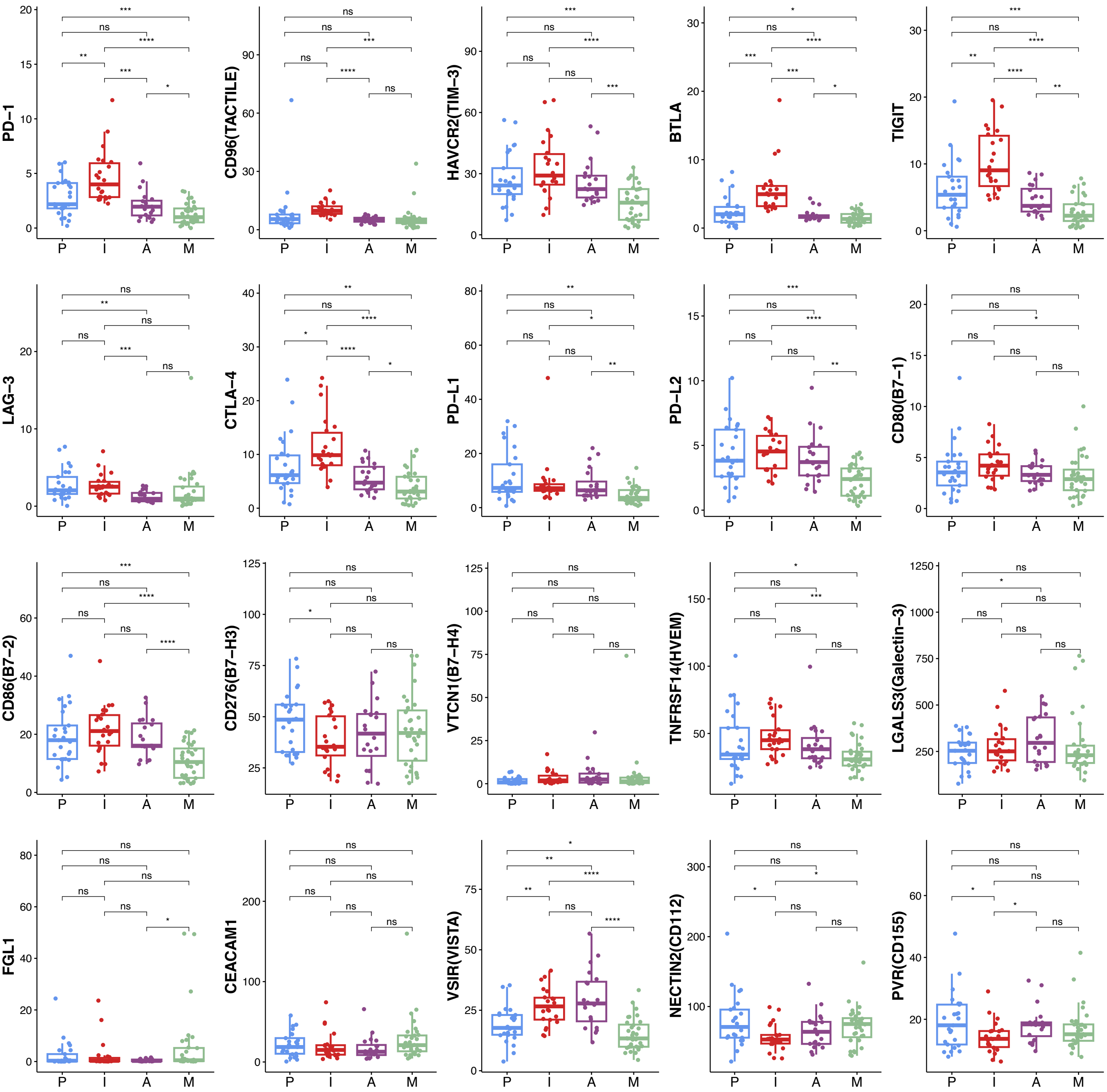

Supplementary Figure 11. Plots of inhibitory immune checkpoint receptors and ligands, and correlation vectors of molecules.

The first seven molecules are immune checkpoint receptors (IRs), and the next thirteen molecules are immune checkpoint ligands in RNAseq data. Most of the IRs were upregulated in the immune-high (I) subgroup which featured high levels of infiltration by immune cells, but the distribution of ligands was not similar to that of the receptors.

Supplementary Figure 12

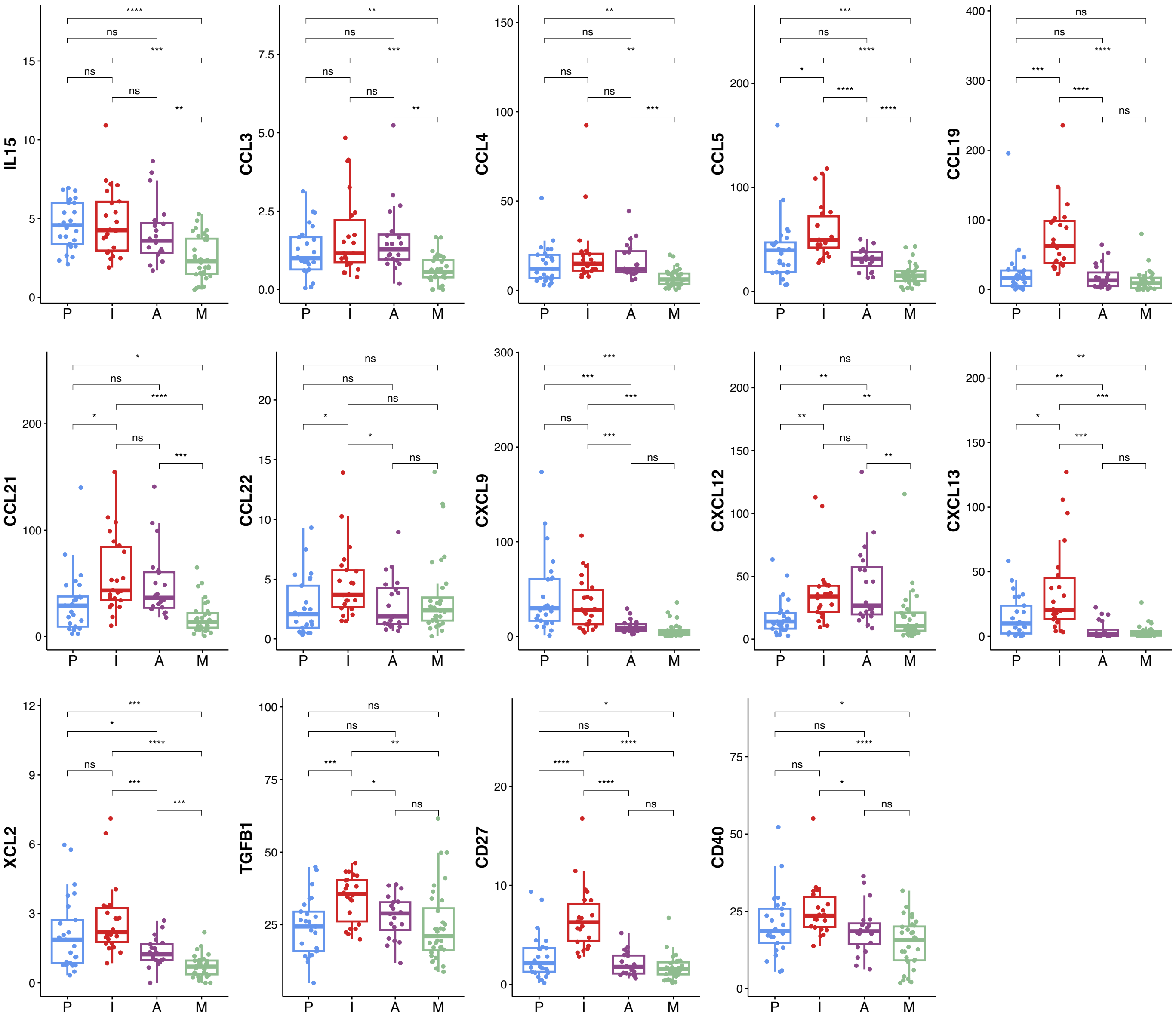

Supplementary Figure 12. Boxplots of cytokines and chemokines and scatter plots for correlation vectors of molecules.

Among the total 50 cytokines and chemokines, fifteen statistically significant molecules in immune-high (I) subgroup were selected and plotted in the boxplot using RNAseq data (Welch's t-test, $P < 0.001$).

Supplementary Figure 13

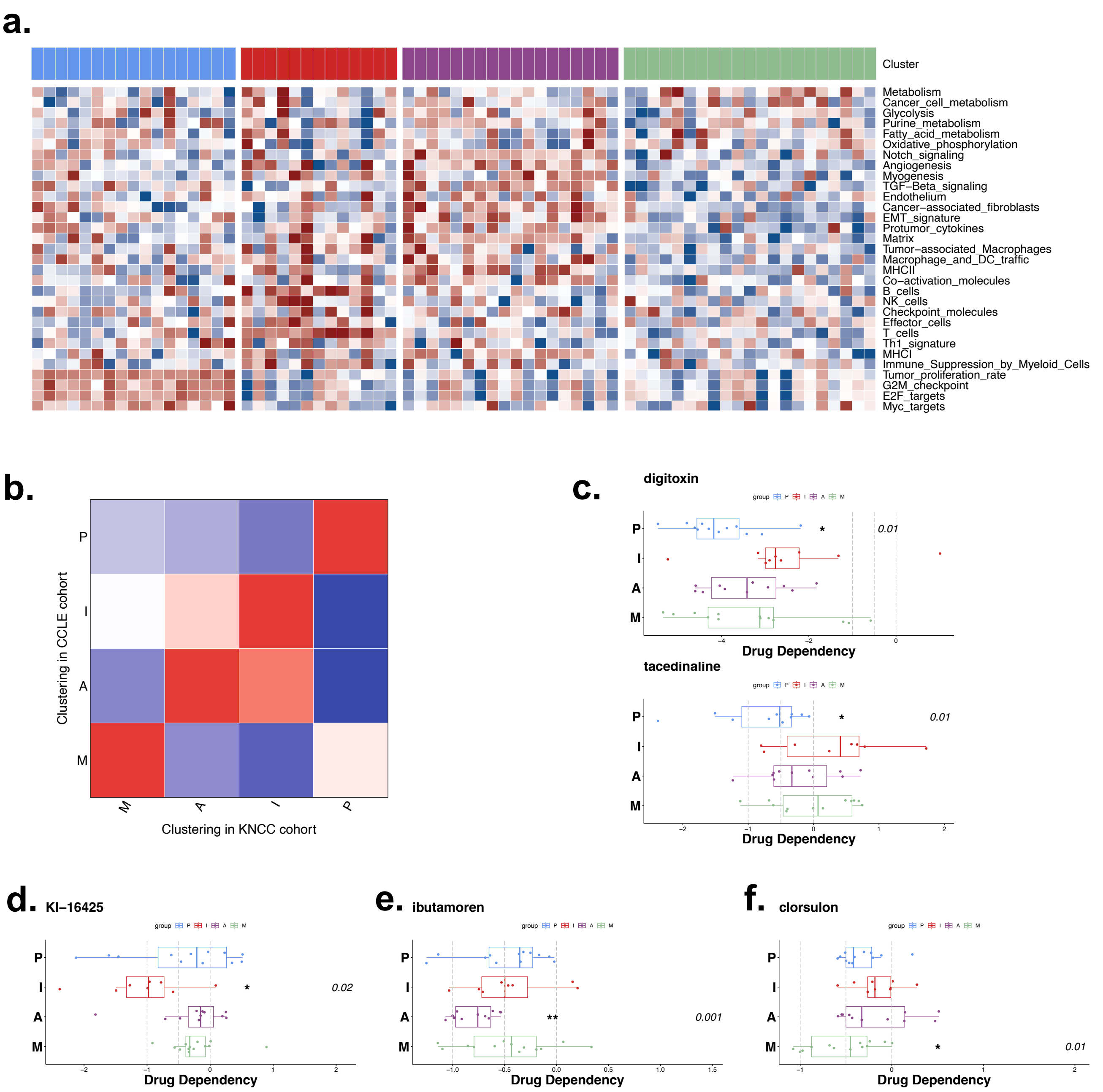

Supplementary Figure 13. Drug sensitivity in association with subgroups

(a) The heatmap illustrates the enrichment of the gene signatures, displaying the clustering of the 69 CCLE LUAD dataset without *EGFR* and *ALK* mutations into four distinct subgroups **(b)** Heatmap depicting relative mean correlation between the P, I, A and M subtypes clustered from KNCC cohort (y-axis) and CCLE cell line cohort (x-axis), respectively (Spearman correlation). **(c-f)** Boxplots depicting drugs exhibiting notably elevated sensitivity exclusively within each subgroup. **(c)** The P subgroup exhibited specific sensitivity when treated with digitoxin and tacedinaline (P = 0.01), **(d)** the I subgroup with KI-16425 (P = 0.02), **(e)** the A subgroup with ibutamoren (P = 0.001) and **(f)** the M subgroup with clorsulon (P = 0.01), exhibiting markedly elevated potential for efficacy.

Supplementary Figure 14

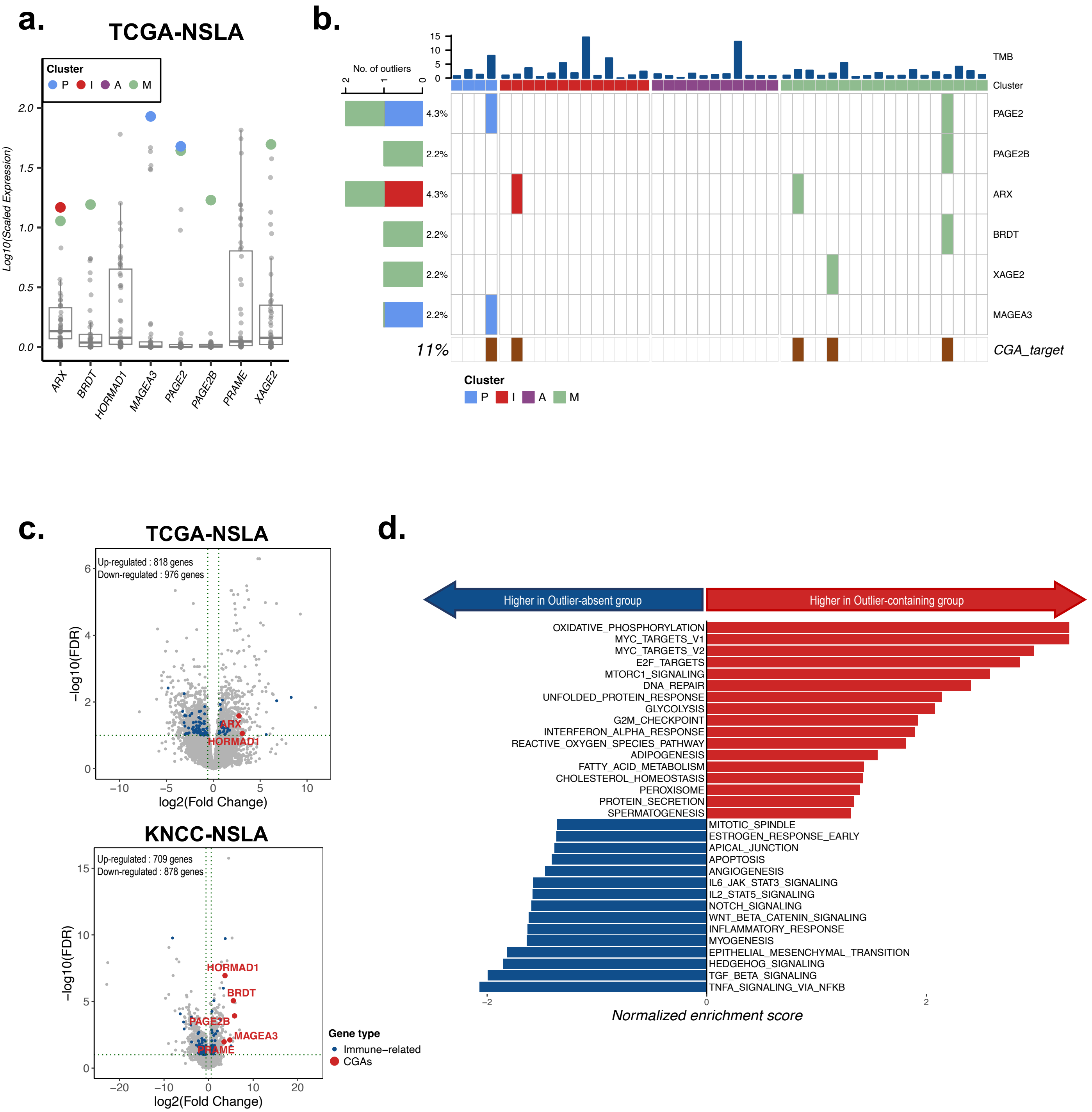

Supplementary Figure 14. Outliers among cancer germline antigens and distribution in TCGA LUAD cohort.

(a) Boxplots of cancer germline antigen expression, log-scaled at the transcriptomic level, depicting outliers by subtypes in never-smoker subgroup of TCGA-LUAD cohort (TCGA-NSLA). **(b)** The heatmap of outlier-containing sample distribution in TCGA-LUAD cohort. **(c)** Volcano plot depicting differential gene expression comparing samples with atypical cancer germline antigen expression in TCGA-NSLA cohort (top) and KNCC-NSLA cohort (bottom). The number of genes up-regulated and down-regulated in outlier-containing group was 327 and 348, respectively in TCGA-NSLA cohort and 709 and 878, respectively in KNCC-NSLA cohort (log2 fold change > 1.5, adjusted p-value < 0.1). **(d)** Pathway enrichment analysis using Hallmark gene sets from MSigDB, associated with outlier cancer germline antigen expression group.

Supplementary Figure 15

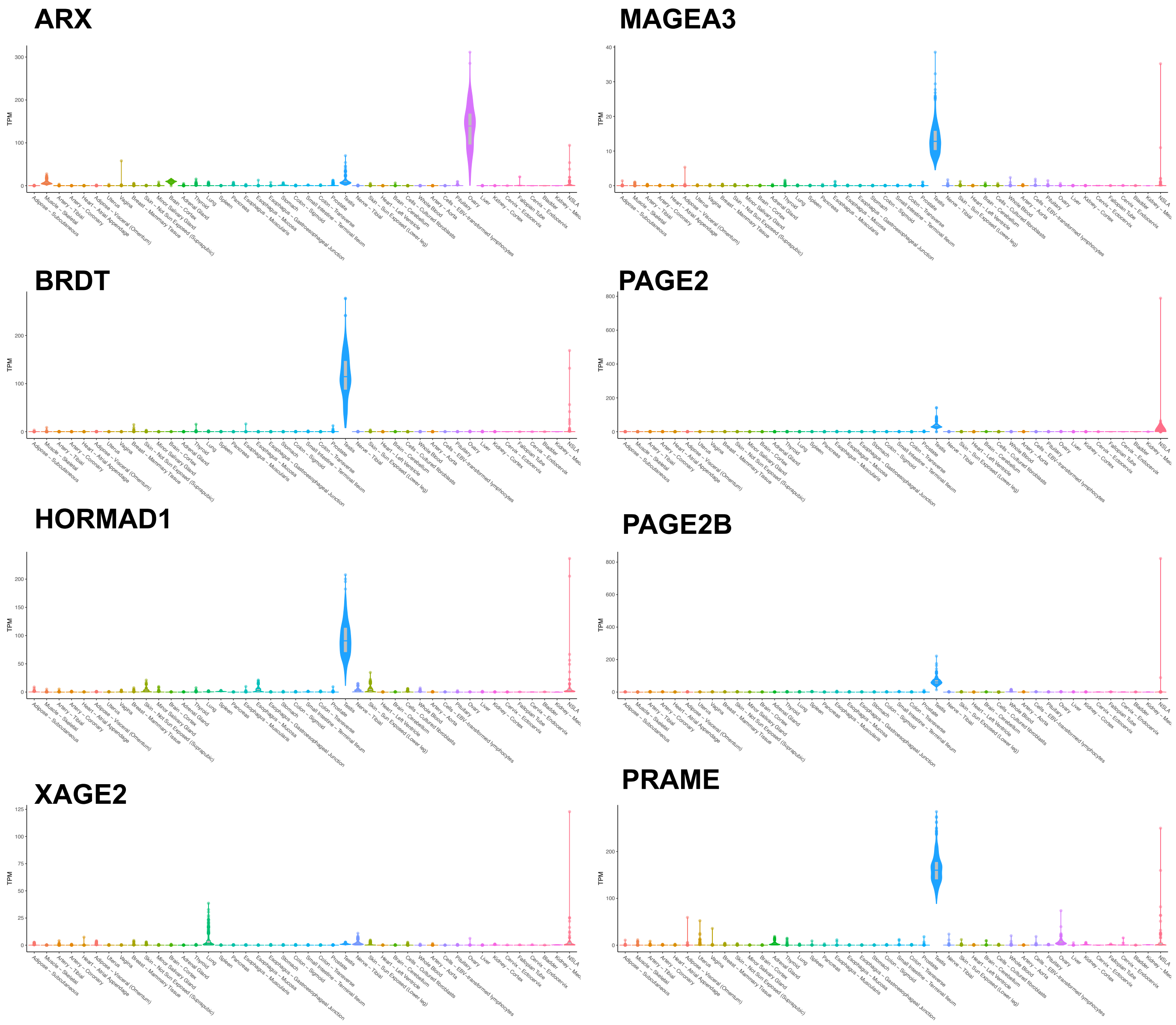

Supplementary Figure 15. Comparison of chosen CGAs outlier distribution in normal cells and NSLA cohort.

Violin plots illustrating average CGAs expression in normal cells and NSLA cohort. Normal tissue data were obtained from the Genotype-Tissue Expression project (GTEx), the publicly accessible data. Mean and 95% confidence interval range of 8 CGAs in NSLA cohort is located on the far right.
